# Do proton pump inhibitors affect the effectiveness of cyclin-dependent kinase 4/6 inhibitors in advanced HR positive, HER2 negative breast cancer? A meta-analysis

**DOI:** 10.3389/fphar.2024.1352224

**Published:** 2024-05-06

**Authors:** Francisco Cezar Aquino de Moraes, Caroline R. M. Pereira, Vitor Kendi Tsuchiya Sano, Estella Aparecida De Laia, Carlos Stecca, Rommel Mario Rodríguez Burbano

**Affiliations:** ^1^ Department of Medicine, Federal University of Pará, Belém, Pará, Brazil; ^2^ Department of Medicine, State University of Rio de Janeiro (UERJ), Rio de Janeiro, Brazil; ^3^ Department of Medicine, Federal University of Acre, Rio Branco, Acre, Brazil; ^4^ Department of Medicine, Fluminense Federal University, Rio de Janeiro, Brazil; ^5^ Mackenzie Evangelical University Hospital, Curitiba, Paraná, Brazil; ^6^ Ophir Loyola Hospital, Belém, PA, Brazil

**Keywords:** CDK 4/6 inhibitors, proton pump inhibitors, breast cancer, hormone receptor-positive, human epidermal growth factor receptor 2-negative

## Abstract

**Background:**

The CDK 4/6 inhibitors, including palbociclib and ribociclib, are the standard first-line treatment for hormone receptor-positive (HR+) and human epidermal growth factor receptor 2-negative (HER2-) metastatic breast cancer. Proton pump inhibitors are one of the most globally prescribed types of medications as part of the treatment for gastroesophageal reflux and heartburn complaints. Medication interactions have been demonstrated, leading to a decrease in the effectiveness of chemotherapy drugs such as capecitabine and pazopanib. However, their role and interaction with targeted therapies such as CDK inhibitors are still poorly understood.

**Methods:**

We searched PubMed, Embase and Web of Science databases for studies that investigated the use of PPI with CDK 4/6 inhibitors versus CDK4/6 alone for advanced or metastatic breast cancer. We systematically searched for the currently available CDK inhibitors: palbociclib, ribociclib and abemaciclib. We computed hazard ratios (HRs), with 95% confidence intervals (CIs). We used DerSimonian and Laird random-effect models for all endpoints. Heterogeneity was assessed using I^2^ statistics. R, version 4.2.3, was used for statistical analyses.

**Results:**

A total of 2,737 patients with advanced breast cancer in 9 studies were included, with six studies described the status menopausal as 217 (7.9%) pre-menopause and 1851 (67.6%) post-menopause, for endocrine sensitivity only five studies described1489 (54.4%) patients were endocrine-sensitive and 498 (182%) endocrine-resistent, 910 (33.2%) patients used PPIs. The overall Progression-Free Survival was in favor of the PPI non-users (HR 2.0901; 95% CI 1.410–2.9498; *p* < 0.001). As well as the subgroup taking palbociclib, revealing statistical relevance for the PPI non-users (HR 2.2539; 95% CI 1.3213–3.8446; *p* = 0.003) and ribociclib subgroup with a slight decrease in hazard ratio (HR 1.74 95% CI 1.02–2.97; *p* = 0.04; I^2^ = 40%). In the multivariate analysis, there was no statistical signifance with ECOG (HR 0.9081; 95% CI 0.4978–16566; p 0.753) and Age (HR 1.2772; 95% CI 0.8790–1.8559; *p* = 0.199). Either, the univariate analysis did not show statistical significance.

**Conclusion:**

Women with HR+ and HER2-advanced metastatic breast undergoing treatment with targeted therapies, specifically CDK 4/6 inhibitors, should be monitored for the use of proton pump inhibitors. Therefore, the use of PPIs should be discussed, weighing the advantages and disadvantages for specific cases. It should be individualized based on the necessity in clinical practice for these cases.

**Systematic Review Registration:**

identifier CRD42023484755

## Graphical Abstract

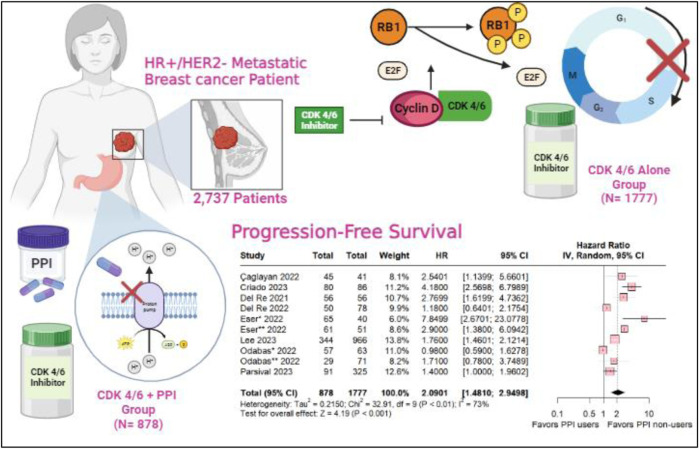

## 1 Introduction

Hormone receptor-positive (HR+) and human epidermal growth factor receptor-2 negative (HER2-) breast cancers collectively account for 80% of all molecular subtypes of malignant neoplasms in the breast (Orrantia-Borunda et al., 2022; Jin et al., 2023; Torrisi et al., 2023). Recent therapeutic advances have significantly contributed to extending survival rates in this patient population, know to harbor a more favorable molecular profile compared to other subtypes. Stage II disease now boasts a 5-year survival rate exceeding 90%, while stage III and stage IV exhibit rates of 72% and 22%, respectively (Cuyún Carter et al., 2021; Jerzak et al., 2023).

Cyclin-Dependent Kinase (CDK) 4/6 inhibitors, including abemaciclib, palbociclib and ribociclib constitute important components of the current standard first-line treatment for patients with HR+/HER2-metastatic breast cancer (mBC) (Chagaleti et al., 2023; Chang and Lam, 2023; Geisler et al., 2023). The combination of Palbociclib with aromatase inhibitors or fulvestrant has emerged as a pivotal advancement, reshaping the treatment paradigms for HR+/HER2-mBC (Bilgin et al., 2017). Notably, in its approval by the Food and Drug Administration (FDA), it was reported that the administration of Proton Pump Inhibitors (PPI) led to a noteworthy 62% reduction in the area under the plasma concentration-time curve (AUC) (Shin and Sachs, 2008; Numico et al., 2017; Hunter et al., 2023).

PPIs are frequently used in cancer patients, with a prevalence ranging from 20% to 55%, aimed at alleviating gastrointestinal symptoms associated with antineoplastic drugs, such as gastroesophageal reflux disease (Targownik et al., 2007; Smelick et al., 2013; Raoul et al., 2021). The advent of new targeted oral therapies for breast cancer has significantly altered the disease´s natural history. However, challenges arise due to differences in drug absorption between periods of fasting and during meals, as well as the sensitivity of target drugs to pH-dependent solubility. These challenges pose unique scenarios for treatment, which was not the case with standard intravenous chemotherapy (Budha et al., 2012; Mullin and Schrogie, 2013; Wedemeyer and Blume, 2014).

The consideration of drug-drug interaction (DDI) is crucial when evaluating potential causes of therapeutic failure in cancer patients (Budha et al., 2012). PPIs reduce gastric acid secretion by irreversibly binding to the adenosine triphosphatase hydrogen-potassium pump in the parietal cell membrane of the stomach (Smelick et al., 2013; Wedemeyer and Blume, 2014). This interference may affect bioavailability and pharmacokinetics of oral anticancer drugs, particularly those classified as weak bases (Chu et al., 2017; Raoul et al., 2023). Considering that more than half of the oral agents used against cancer fall into that category, the efficacy of these drugs may be compromised among PPI users (Riechelmann and Krzyzanowska, 2019).

Studies have shown that the absorption of palbociclib is highly dependent on gastric pH. Similarly, food intake can influence the absorption of abemaciclib, possibly due to alterations in gastric pH caused by proton pump inhibitors (Bellet et al., 2019; Roncato et al., 2020). In contrast, a ribociclib study demonstrated that gastric pH changes do not affect its bioavailability (Samant et al., 2018). In this systematic review and meta-analysis, we aim to clarify the influence of the use of PPIs on progression-free survival (PFS) in patients with HR-positive, ERBB2-negative mBC treated with CDK 4/6 inhibitors (included palbociclib, ribociclib and abemciclib).

## 2 Methods

### 2.1 Protocol and registration

This systematic review followed the Preferred Reporting Items for Systematic Reviews and Meta-Analysis (PRISMA) guidelines (Page et al., 2021) (PRISMA Checklist, Supplementary Tables S1, S2). The protocol was registered in the International Prospective Register of Systematic Reviews (PROSPERO), National Institute for Health and Care Research (NIHR), with registration number CRD42023484755.

We selected the studies based on the PECO question, including studies with women with advanced/metastatic HR+/HER2-breast cancer (P-People) being treated with CDK 4/6 inhibitors (included palbociclib, ribociclib and abemciclib) who used PPIs (E-Exposure) or not (C-Control), to find out whether there is an association between this and the effectiveness of the treatment (O-Outcome).

### 2.2 Eligibility criteria

Studies that met the following eligibility criteria were included: (1) studies of women with HR+/HER2-breast cancer; aged (2) ≥18 years; (3) with advanced-stage or metastatic disease that was not amenable to curative therapy; (4) patients with an Eastern Cooperative Oncology Group (ECOG) performance status score of 0, 1, or 2; (5) who were treated with CDK 4/6 inhibitors; and (3) in which the use of PPIs was described in a group versus CDK 4/6 without PPIs. We excluded studies with overlapping populations, non-randomized clinical trials, and studies without results of interest. The inclusion criteria for each study are listed in Supplementary Table S3.

Therefore, we sought to answer the following question: Can the use of PPIs affect the treatment with CDK 4/6 inhibitors in women with HR+/HER2-breast cancer?

### 2.3 Search strategy

Pubmed, Embase and Web of Science were systematically searched on Octobre 02, 2023. The search strategy with the MeSH terms is detailed in the Supplementary Table S3; Supplementary Material. Aiming the inclusion of additional studies, the references of the included articles and systematic reviews of the literature were evaluated and an alert was established for notifications in each database, in case a study corresponding to the consultation carried out was eventually published. Those found in the databases and in the references of the articles were incorporated into the reference management software (EndNote^®^, version X7, Thomson Reuters, Philadelphia, United States). Duplicate articles were automatically and manually excluded. Titles and abstracts of articles found in the databases were analyzed independently by two reviewers (V.K.T.S. and E.A.L.). Disagreements were resolved by consensus between the senior author (R.M.R.B).

### 2.4 Data extraction

The following baseline characteristics were extracted: (1) study design; (2) details of the regimen in the experimental arm and the control arm according to the CDK4/6 inhibitor used; (5) number of patients assigned to each arm; (6) type of PPI used; (7) age (8) ECOG; (9) menopausal status; (9) resistance or sensitivity to endocrine therapy; and (10) sites of metastases. The search strategy (see Supplementary Table S4) included abemaciclib, a CDK4/6 inhibitor approved in September 2017. However, no studies have identified this drug used in combination with a PPI.

The following outcomes of interest were extracted: PFS, defined as the time elapsed from patient randomization to disease progression or death from any cause (Gyawali et al., 2022), including this outcome for (1) the general population using any type of CDK 4/6 inhibitors, (2) the subgroup using only palbociclib, and (3) the subgroup using only ribiciclib. Two authors (C.R.M.P. and E.A.L.) collected pre-specified baseline characteristics and outcome data.

Where available, the full protocol of each study was consulted to verify study objectives, population, and other relevant information regarding study design and conduction. For publications reporting results from the same study, the most recent or complete publication reporting the information of interest was considered.

### 2.5 Endpoints and subgroup analysis

Outcomes of interest were: (1) PFS for the general population; (2) for women who used only palbociclib and (3) patients who used only ribociclib.

In addition, we performed subgroup analyses for PFS. In the univariate analysis, we evaluated the association with (1) CDK 4/6 dose reduction, (2) metastasis site, (3) visceral/non-visceral disease, and (4) pre/post menopausal status. In the multivariate analysis, we evaluated the association with (1) ECOG and according to (2) the age of the patients included.

### 2.6 Risk of bias assessment

The quality assessment of observational studies was performed using the Newcastle–Ottawa Scale (NOS), in which studies are scored on a 0 to 9 scale according to selection, comparability, and exposure criteria (McPheeters et al., 2012; Ottawa Hospital Research Institute, 2023). Three authors (C.R.M.P., V.K.T.S, and F.C.A.M.) independently conducted the risk of bias assessment and disagreements were resolved by consensus. Funnel-plot analyzes were employed to examine publication bias (Chapter 13: Assessing risk of bias due to missing results in a synthesis, 2023).

### 2.7 Statistical analysis

Hazard ratio (HR) was used to analyze the PFS. We consider HR > 1 favoring the control (PPI non-users) group and HR < 1 favoring the intervention group (PPI-users). The Cochrane *Q*-test and I^2^ statistics were used to assess heterogeneity; *p* values > 0.10 and I^2^ values > 25% were considered to indicate significance for heterogeneity (Higgins et al., 2003). The Sidik-Jonkman estimator was used to calculate the tau^2^ variance between studies (IntHout et al., 2014). We used DerSimonian and Laird random-effect models for all endpoints (DerSimonian and Laird, 1986). Publication bias was assessed using Egger’s linear regression test, the funnel plot, which depicts the relationship between study size and effect size, can be visually inspected for asymmetry in the disperson of studies. A symmetrical funnel plot suggests low heterogeneity in the analysis, while asymmetry may indicate publication bias or a limited number of studies included (Egger et al., 1997). Statistical analyses were performed using R statistical software, version 4.2.3 (R Foundation for Statistical Computing).

## 3 Results

### 3.1 Search results and characteristics of included studies

**FIGURE 1:**
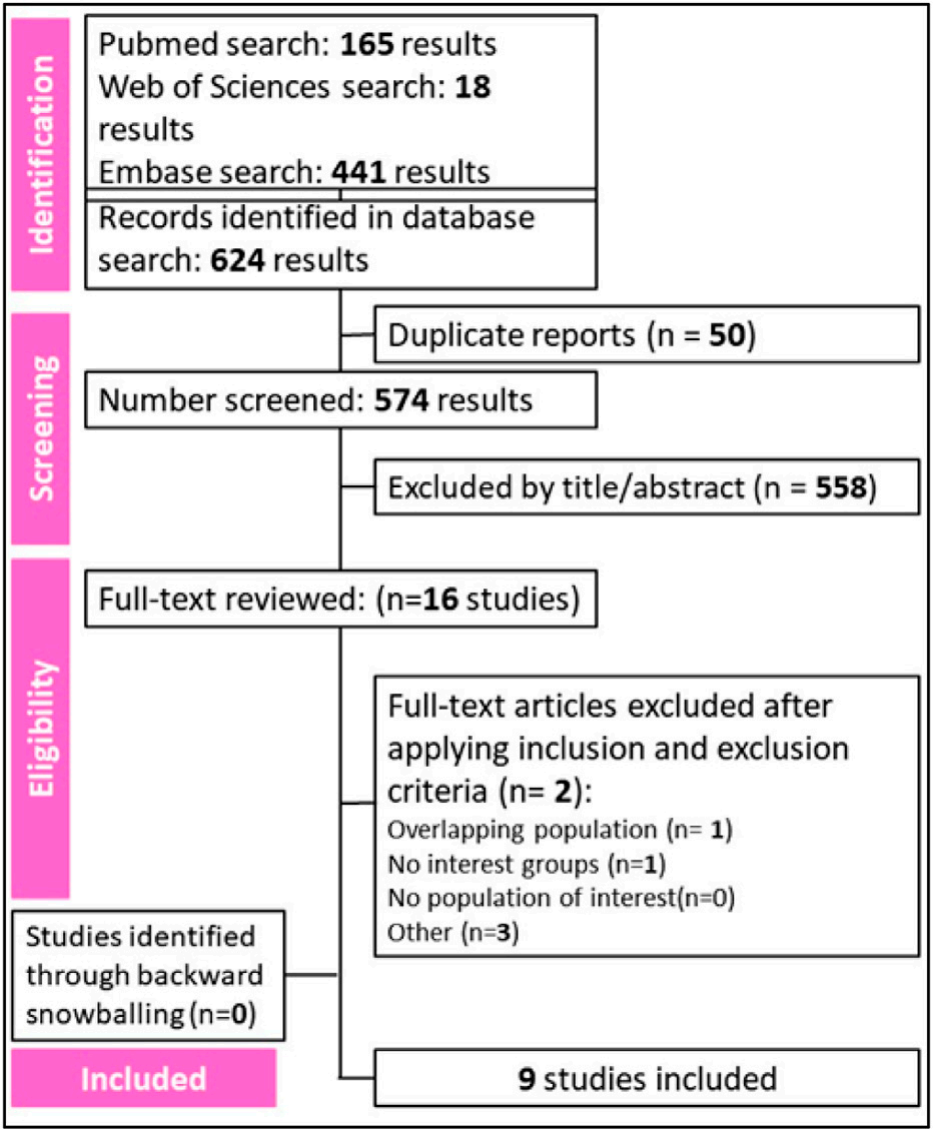
Preferred Reporting Items for Systematic Review and Meta-Analysis (PRISMA) flow diagram of study screening and selection.

A total of 574 references were retrieved in our systematic search. After the removal of duplicate records, and the assessment of the studies based on title and abstract, 558 references were excluded and 16 full-text manuscripts were eligible and thoroughly reviewed for inclusion and exclusion criteria. Of these, 9 studies satisfied the eligibility criteria and formed the scope of the analysis, involving a total of 2737 patients (Del Re et al., 2021; 2022; Eser et al., 2022; Cosimo et al., 2023; ESMO Congress OncologyPRO, 2023; Lee et al., 2023; Odabas et al., 2023; Schieber et al., 2023; Çağlayan et al., 2023). The process of study selection is visually represented in Figure 1 of the PRISMA flow chart.

We included 2,737 patients, of whom 910 (33.2%) used PPIs. Among the patients, 1,489 (54.4%) were sensitive to the endocrine system and 498 (18.2%) were resistant. Additionaly, 217 (7.9%) were pre-menopausal and 1851 (67.6%) were postmenopausal. Table 1 shows the baseline characteristics of the patients included in our meta-analysis.

**TABLE 1:** Design and characterístics of studies included in the meta-analysis.

Author (year)	No. of patients user | No users	Age^a^ user | No users	ECOG status, no (%) user | No users	Menopause status user | No users	Treatment	Disease site user | No users	Endocrine-sensitive or -resistant disease user | No users	PPI user
Del Re et al. (2021)	56 | 56	NA	0–40 (71.4%); 1–14 (25%); 2–2 (3.6%)	Pre: 8 (14.3%); Post 48 (85.7)	Palbociclib	Visceral 24 (42.9%); non visceral 32 (57.1%)	Sensitive 36 (64.3%); resistant 20 (35.7%)	Lansoprazole 42 (37.5%); Omeprazole 11 (9.8%); Pantoprazole 2 (1.8%); Esomeprazole 1 (0.9%)
			0–44 (78.6%); 1–11 (19.6%); 2–1 (1.8%)	Pre: 11 (19.6%); Post 45 (80.4)		Visceral 31 (55.4%); non visceral 25 (44.6%)	Sensitive 35 (62.5%); resistant 21 (37.5%)	
Del Re et al. (2022)	50 | 78	NA	0–32 (64%); 1–15 (30%); 2–3 (6%)	Pre: 13 (26%); Post 37 (74%)	Ribociclib	Visceral 26 (52%); Non visceral 24 (48%)	Sensitive 44 (88%); resistant 6 (12%)	Lansoprazole 68%); Omeprazole 6 (12%); Pantoprazole 7 (14%); Esomeprazole 3 (6%)
			0–62 (79.5%); 1–11 (14.1%); 2–5 (6.4%)	Pre: 18 (23.1%); Post 60 (76.9%)		Visceral 41 (52.6%); Non visceral 37 (47.4%)	Sensitive 62 (79.5%); resistant 16 (20.5%)	
Eser et al. (2022)	126 | 91	Palbociclib group: 60.47 ± 10.86 Ribociclib group: 57.85 ± 10.56	0–26 (20.6%); 1–80 (63,5%); 2–20 (15,9%)	Pre: 39 (30.9%); Post 87 (69.1%)	Ribociclib; Palbociclib	Visceral 71 (61.2%), Non visceral 45 (38.8%)	Sensitive 58 (46%), Resistent 68 (64%)	Palbociclib group: Lansoprazole 9 (13.8%); Omeprazole 3 (4.6%); Pantoprazole 28 (43.1%); Esomeprazole 8 (12.3%); Rabeprazole 17 (26.2%)Ribociclib group: Lansoprazole 3 (4.9%); Omeprazole 12 (19.7%); Pantoprazole 18 (29.5%); Esomeprazole 18 (29.5%); Rabeprazole 10 (16.4%)
		Palbociclib group: 57.49 ± 9.5; Ribociclib group: 50.81 ± 12.21	0–31 (34%); 1–54 (59.3%); 2–6 (6.7%)	Pre: 36 (39.6%); Post 55 (60.4%)		Visceral 46 (50.5%), Non visceral 45 (49.5%)	Sensitive 57 (62.7%), Resistent 34 (37.3%)	
Schielber et al. (2023)	32 | 50	62.04 ± 3.62/67.29 ± 4.45	0–11 (34.4%); 1–16 (50%); 2+- 5 (15.6%)	Pre: 5 (15.6%); Post 27 (84.5)/Pre:12 (24%); Post 38 (76)	Palbociclib	Non visceral 11 (34.4%)/Non visceral 29 (58%)	NA	Pantoprazole 9 (28.1) Omeprazole 21 (65.6) Lansoprazole 1 (3.1) Esomeprazole 1 (3.1)
			0–25 (50%); 1–17 (34%); 2+- 8 (16%)					
Lee et al. (2023)	344 | 966	≤50 53 (15.4); >50,291 (84.6)	NA	Pre 6 (1.7%); Post 338 (98.3%)	Palbociclib	Vicsceral: 68 (19,8%), Non visceral 104 (30.2%); Other sites 23 (6.7%)	Sensitive 292 (84.9%); resistant 52 (15.1%)	NA
		≤50,149 (15.4); >50,817 (84.6)		Pre 15 (1.6%); Post 951 (98.4%)		Visceral 238 (24.7); Non visceral 264 (27.3%); Other sites 67 (6.9%)	Sensitive 819 (84.8%); resistant 147 (15.2%)	
Odabas et al. (2023)	57 | 63	60.40 ± 12.86	0–35 (55.6%); 1–20 (31.7%); 2/3–8 (12.7%)	Pre: 19 (30.6); Post: 43 (69.4) Pre: 8 (14.0); Post: 49 (86.0) Unknown: 1 (1.65)	Palbociclib; Ribociclib	Visceral: 30 (47.6); Non visceral: 33 (52.4)	Sensitive: 24 (38.1); Resistant:39 (61.9)	Lansoprazole 17 (29.8) Pantoprazole 14 (24.6) Esomeprazole 16 (28.1) Rabeprazole 5 (8.8) Omeprazole 5 (8.8)
		54.22 ± 13.08	0–33 (57.9); 1–20 (35.1); 2/3–4 (7.0)			Visceral: 29 (50.9); Non visceral: 28 (49.1)	Sensitive: 30 (52.6); Resistant:27 (47.4)	
Odabas et al. (2023)	29 | 71	59.75 ± 12.33	0–30 (42.3); 1–37 (52.1); 2/3–4 (5.6)	Pre: 22 (31.0); Post: 49 (69.0)/Pre: 5 (17.2); Post: 24 (82.8)	Palbociclib; Ribociclib	Visceral: 33 (46.5); Non visceral: 38 (53.5)	Sensitive: 23 (32.4); Resistant: 48 (67.6)	Lansoprazole 14 (48.3) Pantoprazole 7 (24.1) Esomeprazole 4 (13.8) Rabeprazole 3 (10.3) Omeprazole 1 (3.4)
		52.49 ± 10.31	0–16 (55.2); 1–11 (37.9); 2–3 - 2 (6.9)			Visceral: 16 (55.2); Non visceral: 13 (44.8)	Sensitive:9 (31.0); Resistant: 20 (69.0)	
Çaglayan et al. (2022)	45 | 41	55 ± 11.8 56 ± 13.9	NA	NA	Palbociclib; Ribociclib	NA	NA	Pantoprozole Lansoprozole Esomeprozole Rabeprozole
Criado et al. (2023)	80 | 86	NA	NA	NA	Palbociclib	NA	NA	NA
Cosimo et al. (2023)	91 | 325	NA	NA	NA	Palbociclib	NA	NA	NA

^a^ Median range, years; ECOG, eastern cooperative oncology group; NA, not available Progression-Free Survival.

#### 3.1.1 Overall population

**FIGURE 2:**
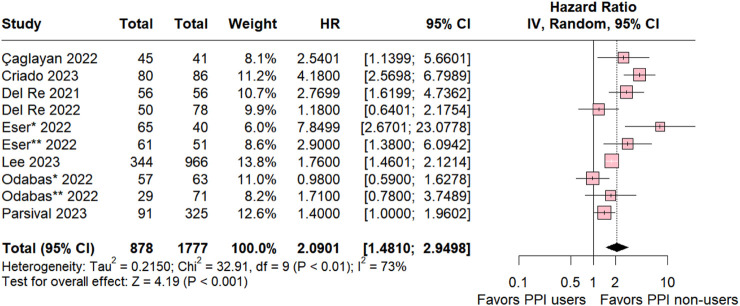
Progression-free survival overall of patients with breast cancer treated with PPI users and PPI non-users.

A comprehensive comparative analysis was conducted across the nine included studies investigating Progression-Free Survival (PFS) in patients concurrently using proton pump inhibitors (PPIs) and those in a control group without this medication. The results reveal a significant association between non-use of PPIs and a higher likelihood of PFS (HR 2.0901; 95% CI 1.410–2.9498; *p* < 0.001; I^2^ 73%; Figure 2), accompanied by notable heterogeneity among the studies. These findings highlight the potential impact of abstaining from PPIs on progression-free survival.

#### 3.1.2 Palbociclib subgroup

**FIGURE 3:**
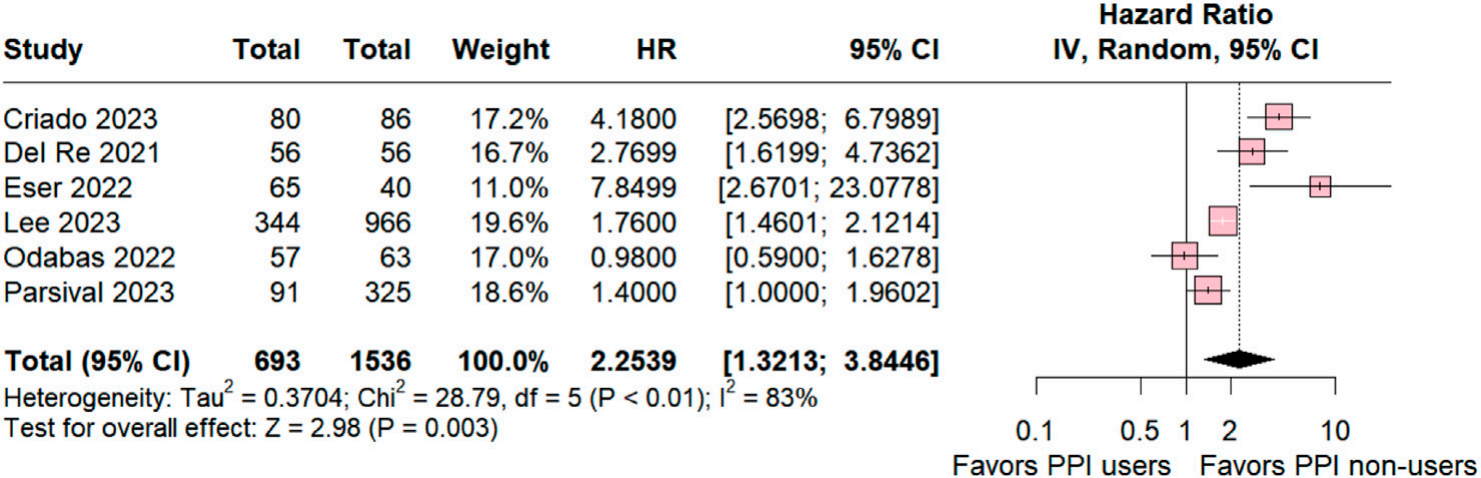
Progression-free survival overall of patients with breast cancer taking palbociclib treated with PPI users and PPI non-users.

The subgroup analysis of individuals taking palbociclib was conducted for six studies, revealing statistical relevance for the control group (HR 2.2539; 95% CI 1.3213–3.8446; *p* = 0.003; I^2^ 83%; Figure 3). The analysis also demonstrated significant heterogeneity among the studies, suggesting variation among the studies included in this analysis.

#### 3.1.3 Ribociclib subgroup

**FIGURE 4:**
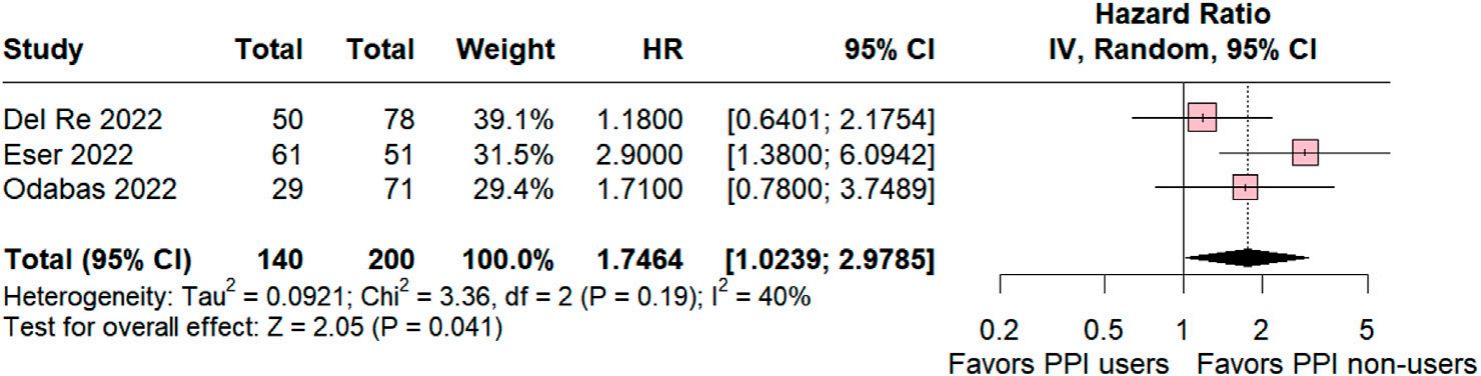
Progression-free survival overall of patients with breast cancer taking ribociclib treated with PPI users and PPI non-users.

In the ribociclib subgroup the estimated PFS included three studies was significant in favor for PPI non-users compared with PPI users (HR 1.74 95% CI 1.02–2.97; *p* = 0.04; I^2^ = 40% Figure 4). The value of I^2^ demonstrated a slight heterogeneity rate among the studies, which was expected because the studies were retrospective observational studies with variations.

#### 3.1.4 Univariate analysis

The univariate analysis included the variables CDK inhibitor dose reduction (HR 0.9352; 95% CI 0.7138–1.2254; p 0.627; I^2^ 0%; Supplementary Figure S1A), metastasis sites (HR 1.1575; 95% CI 0.9554–1.4024; p 0.135; I^2^ 41%; Supplementary Figure S1B), visceral/non-visceral disease (HR 0.8757; 95% CI 0.5607–1.3678; p 0.560; I^2^ 63%; Supplementary Figure S1C) and pre/pos menopause (HR 1.0782; 95% CI 0.6890–1.6872; p 0.742; I^2^ 42%; Supplementary Figure S1D) all variables did not show statistical significance in the univariate analysis.

#### 3.1.5 Multivariate analysis

The multivariate analysis included three studies with ECOG and PFS not show statistical significance (HR 0.9081; 95% CI 0.4978–16566; p 0.753; I^2^ 54%; Supplementary Figure S2A). Furthermore, three studies were included with Age and PFS not demonstrated an association between the variables (HR 1.2772; 95% CI 0.8790–1.8559; p 0.199; I^2^ 60%; Supplementary Figure S2B), besides a significant heterogeneity rate among the studies in both analyses.

#### 3.1.6 Sensitivity analysis and quality assessment

**FIGURE 5:**
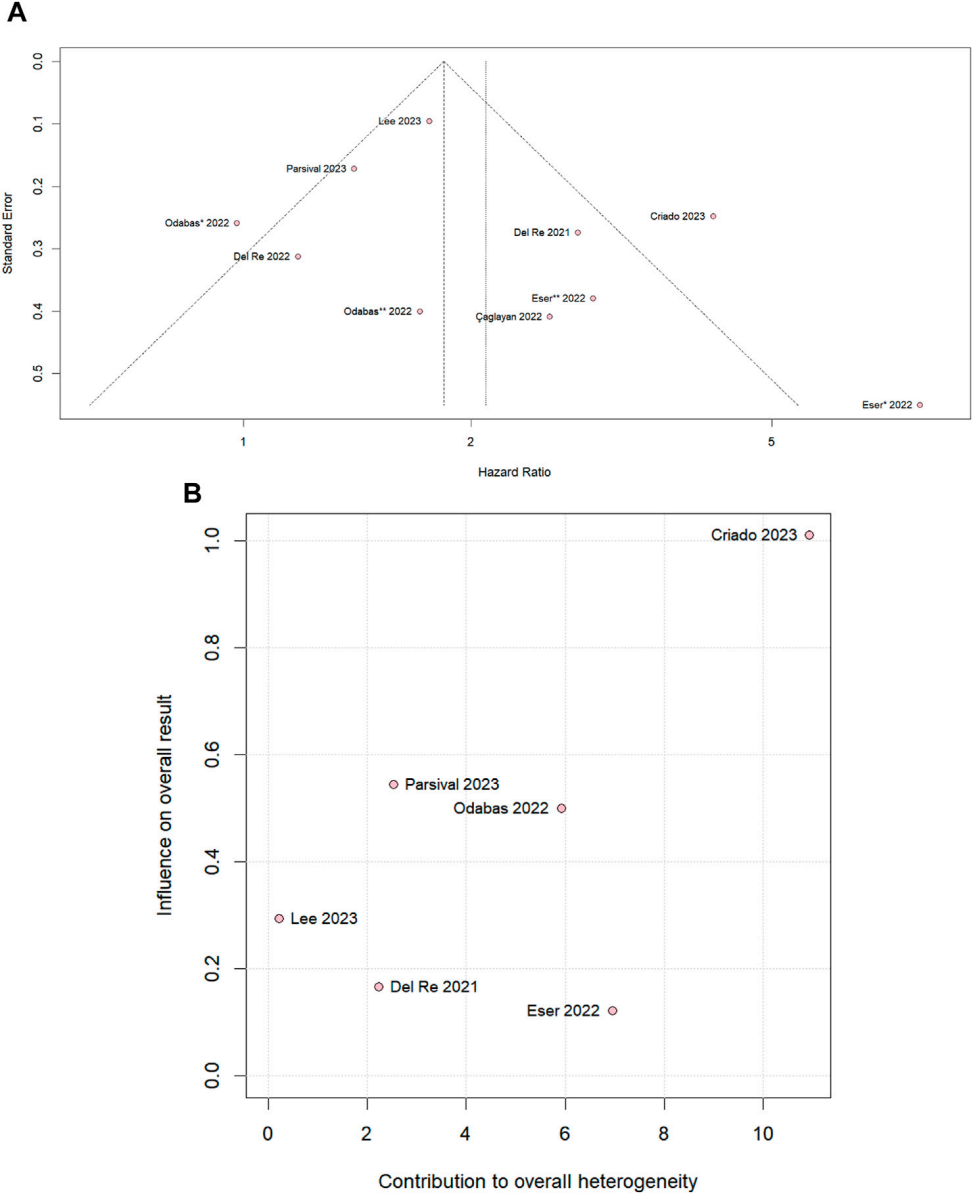
**(A)** Funnel plot for PFS in the overall population and **(B)** diagram of the influence of heterogeneity on the PFS results for the palbociclib subgroup population.

We performed leave-one-out sensitivity analyses for all outcomes. There was no significant difference in PSF in the overall population (Supplementary Figure S3A) and PFS in the palbociclib subgroup (Supplementary Figure S3B), and there was no significant change in the heterogeneity value when studies were omitted. In the PFS ribociclib subgroup (Supplementary Figure S3C), the heterogeneity value, which is I2 40%, becomes 0% by omitting Del Re et al., 2022 and was a significant difference favoring the PPI-nonusers. In addition, omitting Eser et al. (2022), the heterogeneity becomes 0% and a tendency to move toward the control group. The slight asymmetrical distribution against standard errors, which is indicative of a small sample of studies, is represented in the funnel plot of the PFS overall population in Figure 5A. Supplementary Figure S1D shows a diagram of the influence of heterogeneity on the outcomes of the included studies in the PFS overall population that CRIADO 2023 was responsible for the high rate of heterogeneity, even though it is one of the major contributors to the study’s outcome as well as in the PFS palbociclib subgroup (Figure 5B). The funnel of the ribocicblib subgroup (Supplementary Figure S3G) shows a symmetrical distribution of comparable studies but with only three studies and in the diagram (Supplementary Figure S3H) demonstrated that the major contributors for the heterogeneity were Del Re et al. (2022); Eser et al. (2022).

The individual assessment of each study included in the meta-analysis is depicted in Supplementary Figure S6. Whereas five studies were considered as high quality, three studies were considered lower quality with a score ≤7 using the Newcastle-Ottawa Scale.

## 4 Discussion

In this systematic review and meta-analysis encompassing 9 studies and 2,737 patients, we examined the outcomes for women with HR+/HER2-advanced breast cancer undergoing treatment with cyclin-dependent kinase (CDK) 4/6 inhibitors, comparing those who used proton pump inhibitors (PPIs) against those who did not. The analysis of palbociclib and ribociclib subgroups was conducted considering their unique mechanisms of action and interactions with proton pump inhibitors (PPIs), highlighting differences in solubility, metabolism, and pharmacokinetic interactions. This is clinically important for assessing the influence of PPI usage on treatment effectiveness and informing individualized therapy choices for patients with advanced breast cancer.

We used the I^2^ for asses the heterogeneity of analysis, Egger’s test for publications bias by the symmetry of funnel plot and the sensibility analysis to evaluate identified the influential studies in heterogeneity and the robustness of findings.

Our results underscore a significant association between non-use of PPIs and an increased likelihood of progression-free survival (PFS) (HR 2.0901; 95% CI 1.410–2.9498; *p* < 0.001). Subgroup analysis further revealed that the addition of PPIs significantly reduced PFS for patients treated with palbociclib and ribociclib.

CDK 4/6 inhibitors within the CDK4 (INK4)-retinoblastoma (Rb) pathway govern the regulatory phases of the cell cycle, specifically orchestrating progression from G1 (pre-DNA synthesis) to S (DNA synthesis) (Hamilton and Infante, 2016). CDK 4/6 assumes a pivotal role in overseeing the transition from G1 to S through its interaction with D-type cyclins, thereby inducing the phosphorylation of Rb (Wang et al., 2023). Elevated cyclin-dependent kinase 4/6 activity ensues from mutations in CDK 4/6, the depletion of CDK regulators, and the heightened expression of D-type cycling—all converging to instigate the proliferation of cancer cells. CDK 4/6 inhibitors act by attenuating the phosphorylation state of Rb, thereby reducing the concentration of phosphorylated Rb. This reduction creates an environment conducive to the formation of RB-E2F transcription factor complexes, ultimately incapacitating the E2F transcription factors that activate genes essential for the initiation of the S phase and DNA replication (Malumbres and Barbacid, 2009; Wang et al., 2023; Knudsen et al., 2023; Pan et al., 2023).

PPIs represent weak bases characterized by a substituted pyridine with a primary pK ranging from 3.8 to 4.9 coupled with a benzimidazole exhibiting a secondary pKa of approximately 1.0. PPI acts on gastric ATPase through covalent binding (Shin et al., 2006; Srebro et al., 2022). In an acidic environment, they undergo conversion to sulfenic acids or sulfenamides, initiating a reaction that results in the formation of a disulfide bond with a cysteine or multiple cysteines accessible from the luminal surface of ATPase (Proton Pump Inhibitors, 2012). Because of the establishment of disulfide bonds, their inhibitory effects significantly persist longer (Robinson and Horn, 2003).

The interaction between PPIs and antineoplastic drugs is highly variable among cancer patients. Existing studies have indicated that the concurrent use of PPIs can diminish the antitumor effectiveness or certain drugs like capecitabine and pazopanib (van Leeuwen et al., 2014; Raoul et al., 2023). On the other hand, it does not appear to exert a significant influence on the clinical outcomes of patients undergoing treatment with epidermal growth factor receptor (EGFR) inhibitors (Hilton et al., 2013; Kumarakulasinghe et al., 2016; Chu et al., 2017; Moreau-Bachelard et al., 2022).

In our analysis, ta global assessment of patients using CDK 4/6 inhibitors revealed a significant risk of progression associated with the use of PPIs (HR 2.0901; 95% CI; 1.410–2.9498; *p* < 0.001). These findings parallel results observed in advanced small cell lung cancer (SCLC) treated with programmed death-ligand 1 (PD-L1) inhibitors, where concurrent use of PPIs was linked to a 74.9% increased risk of progression (HR 1.749; 95% CI; 1.285–2.380) and a 58.3% increased risk of death (HR = 1.583, 95% CI; 1.059–2.366) (Zhang et al., 2023).

In the palbociclib group, negative outcomes associated with the use of PPIs were maintained (HR 2.2539; 95% CI 1.3213–3.8446; *p* = 0.003; I^2^ 83%), with a slight increase in relative risk compared to the overall group. This aligns with a study by Sun et al. (2017) evaluating the interaction of palbociclib with rabeprazole, a PPI. They found that at pH above 4, rabeprazole decreased palbociclib’s area under the curve (AUC) by 62% and its maximum concentration (Cmax) by 80%. These findings support the potential association between PPI use and reduced oral chemotherapy effectiveness (Sun et al., 2017). However, Sun et al. also demonstrated that taking palbociclib with food significantly mitigated the impact of rabeprazole, restoring AUC and Cmax closer to baseline levels. This suggests that dietary strategies may offer a potential intervention to optimize palbociclib exposure in PPI users (Sun et al., 2017).

In the subgroup analysis involving ribociclib, the hazard ratio significantly favored PPI non-users over PPI users (HR 1.74; 95% CI 1.02–2.97; *p* = 0.04; I^2^ = 40%). However, this ratio is notably lower in comparison to the palbociclib subgroup. This discrepancy can be attributed to the higher solubility of ribociclib at pH levels below 4.5, contrasting with palbociclib, which already shows reduced solubility at or above pH 4 (Samant et al., 2018; Bellet et al., 2019). Another possible explanation lies in the potential interaction between CDK4/6 inhibitors and PPIs, on the CYP2C19 enzyme responsible for metabolizing both substances (Hamilton and Infante, 2016; Imhann et al., 2016; Wang et al., 2023). Studies by Imhann et al. (2016) and Hamilton and Infante (2016) demonstrated that omeprazole inhibits CYP2C19 activity, which was further corroborated by Wang et al. (2023). This inhibition can lead to a significant increase in plasma concentrations of certain CDK4/6 inhibitors, potentially leading to increased efficacy but also raising concerns about drug toxicity. Therefore, further investigation into the specific enzyme interactions and their clinical implications is warranted.

While our study provides valuable insights, certain limitations should be acknowledged. Firstly, the observed high heterogeneity may stem from the diverse types of PPI used, given the variable effectiveness of each (Zhang et al., 2017; He et al., 2022). Unfortunately, due to a lack of detailed data in the studies, we were unable to conduct a specific analysis of the impact of each type of PPI on PFS. Secondly, the absence of data prevented the reporting of additional outcomes of interest, including overall response rate (ORR), complete response (CR), partial response (PR), stable disease (SD), and overall survival (OS). However, it is important to note that these data gaps this did not prevent the drawing of solid conclusions concerning the results analyzed within each group. Further investigations addressing these limitations could provide a more comprehensive understanding of the nuanced relationship between CKD 4/6 inhibitors, PPIs, and varios clinical ouctomes.

## 5 Conclusion

This groundbreaking meta-analysis is the first to examine the impact of PPIs on the efficacy of CDK 4/6 inhibitors in women diagnosed with HR+/HER2-metastatic breast cancer. Our results add significant insights to the existing body of literature, substantiating the notion that PPI usage ay adversely influence the efectiveness of CDK 4/6 inhibitors. This discovery emphasizes the need for careful consideration and potential contraindication of PPIs in this specific clinical context, as their use is strongly correlated with a substantial reduction in PFS among women undergoing treatment with palbociclib or ribociclib. Additionally, our conclusion highlights the urgent need for future research, particularly prospective studies, to rigorously validate these findings and explore underlying mechanisms, crucial for optimizing therapeutic strategies in the treatment of metastatic breast cancer.

## Data Availability

The original contributions presented in the study are included in the article/Supplementary Materials, further inquiries can be directed to the corresponding author.

## Abbreviations

AUC, area under the plasma concentration time curve; Cls, confidence intervals; CDK, cyclin-dependent kinase; CR, complete response; DDI, drug-drug interaction; ECOG, eastern cooperative oncology group; EGFR, epidermal growth factor receptor; FDA, food and drug administration; G1, pre-DNA synthesis; HER2, human epidermal growth factor receptor 2-negative; HR+, hormone receptor-positive; HR, hazard ratio; Mbc, metastatic breast cancer; MTX, methotrexate; ORR, objective response rate; OS, overall survival; SD, stable disease; PD, progressive disease; PD-L1, programmed death ligand 1; PFS, progression-free survival; PPI, proton pump inhibitors; PR, partial response; PRISMA, preferred reporting items for systematic reviews and meta-analysis; PROSPERO, international prospective register of systematic reviews; Rb, retinoblastoma; S, DNA synthesis; TKI, tyrosine kinase inhibitor.
