# Supplementary material for: Do proton pump inhibitors affect the effectiveness of cyclin-dependent kinase 4/6 inhibitors in advanced HR positive, HER2 negative breast cancer? A meta-analysis
Source: Front Pharmacol. 2024 May 6;15:1352224. doi: 10.3389/fphar.2024.1352224 (PMC11102992; doi:10.3389/fphar.2024.1352224)
Supplement: Supplementary file 1 [file DataSheet1.docx]

Supplementary Material

**Supplementary Table 1** PRISMA 2020 Checklist.

**Supplementary Table 2** PRISMA 2020 for Abstract Checklist.

**Supplementary Table 3** Inclusion and exclusion criteria of included studies

**Supplementary Table 4** Search strategies.

**Supplementary Table 5** Treatment regimens from studies included in this systematic review and meta-analysis

**Supplementary Table 6** Quality assessment using the Newcastle-Ottawa Scale (NOS) of include studies in systematic review and meta-analysis.

**Supplementary Figure 1** Univariate Analysis: A- Dose Reduction; B- Metastatic sites; C – Metastasis Visceral or Non-visceral; D- Pre or Post-menopause

**Supplementary Figure 2** Multivariate Analysis: A- ECOG; B – Age;

**Supplementary Figure 3** A- Sensitive PFS Overall; B- Sensitive PFS Palbo; C- Sensitive PFS Ribo; D- Baujart overall PFS; E- Funnel Palbo; F- Funnel Ribo PFS; G- Baujart Ribo PFS

**Table S1. PRISMA 2020 Checklist.**

| Section and Topic | Item # | Checklist item | | Location where item is reported |
| --- | --- | --- | --- | --- |
| TITLE | | | |  |
| Title | 1 | Identify the report as a systematic review. | | Yes |
| ABSTRACT | | | |  |
| Abstract | 2 | See the PRISMA 2020 for Abstracts checklist. | | Supplementary Materials: Table S2. |
| INTRODUCTION | | | |  |
| Rationale | 3 | Describe the rationale for the review in the context of existing knowledge. | | Yes |
| Objectives | 4 | Provide an explicit statement of the objective(s) or question(s) the review addresses. | | Yes |
| METHODS | | | |  |
| Eligibility criteria | 5 | Specify the inclusion and exclusion criteria for the review and how studies were grouped for the syntheses. | | Yes |
| Information sources | 6 | Specify all databases, registers, websites, organisations, reference lists and other sources searched or consulted to identify studies. Specify the date when each source was last searched or consulted. | | Yes |
| Search strategy | 7 | Present the full search strategies for all databases, registers and websites, including any filters and limits used. | | Supplementary Materials: Table S4 Search Strategies |
| Selection process | 8 | Specify the methods used to decide whether a study met the inclusion criteria of the review, including how many reviewers screened each record and each report retrieved, whether they worked independently, and if applicable, details of automation tools used in the process. | | Yes |
| Data collection process | 9 | Specify the methods used to collect data from reports, including how many reviewers collected data from each report, whether they worked independently, any processes for obtaining or confirming data from study investigators, and if applicable, details of automation tools used in the process. | | Yes |
| Data items | 10a | List and define all outcomes for which data were sought. Specify whether all results that were compatible with each outcome domain in each study were sought (e.g. for all measures, time points, analyses), and if not, the methods used to decide which results to collect. | | Yes |
|  | 10b | List and define all other variables for which data were sought (e.g. participant and intervention characteristics, funding sources). Describe any assumptions made about any missing or unclear information. | | Yes |
| Study risk of bias assessment | 11 | Specify the methods used to assess risk of bias in the included studies, including details of the tool(s) used, how many reviewers assessed each study and whether they worked independently, and if applicable, details of automation tools used in the process. | | Yes |
| Effect measures | 12 | Specify for each outcome the effect measure(s) (e.g. risk ratio, mean difference) used in the synthesis or presentation of results. | | Yes |
| Synthesis methods | 13a | Describe the processes used to decide which studies were eligible for each synthesis (e.g. tabulating the study intervention characteristics and comparing against the planned groups for each synthesis (item #5)). | | Yes |
|  | 13b | Describe any methods required to prepare the data for presentation or synthesis, such as handling of missing summary statistics, or data conversions. | | Yes |
|  | 13c | Describe any methods used to tabulate or visually display results of individual studies and syntheses. | | Yes |
|  | 13d | Describe any methods used to synthesize results and provide a rationale for the choice(s). If meta-analysis was performed, describe the model(s), method(s) to identify the presence and extent of statistical heterogeneity, and software package(s) used. | | Yes |
|  | 13e | Describe any methods used to explore possible causes of heterogeneity among study results (e.g. subgroup analysis, meta-regression). | | Yes |
|  | 13f | Describe any sensitivity analyses conducted to assess robustness of the synthesized results. | | Yes |
| Reporting bias assessment | 14 | Describe any methods used to assess risk of bias due to missing results in a synthesis (arising from reporting biases). | | Yes |
| Certainty assessment | 15 | Describe any methods used to assess certainty (or confidence) in the body of evidence for an outcome. | | Yes |
| RESULTS | | | |  |
| Study selection | 16a | Describe the results of the search and selection process, from the number of records identified in the search to the number of studies included in the review, ideally using a flow diagram. | | Yes  Figure 1 |
|  | 16b | Cite studies that might appear to meet the inclusion criteria, but which were excluded, and explain why they were excluded. | Yes | |
| Study characteristics | 17 | Cite each included study and present its characteristics. | | Yes  Table 1 |
| Risk of bias in studies | 18 | Present assessments of risk of bias for each included study. | | Supplementary Materials: Figure 4. |
| Results of individual studies | 19 | For all outcomes, present, for each study: (a) summary statistics for each group (where appropriate) and (b) an effect estimate and its precision (e.g. confidence/credible interval), ideally using structured tables or plots. | | Yes  Figure 2-4 |
| Results of syntheses | 20a | For each synthesis, briefly summarise the characteristics and risk of bias among contributing studies. | | Yes Supplementary Materials: Figure 1 and 2 |
|  | 20b | Present results of all statistical syntheses conducted. If meta-analysis was done, present for each the summary estimate and its precision (e.g. confidence/credible interval) and measures of statistical heterogeneity. If comparing groups, describe the direction of the effect. | | Yes  Figure 2, 3, 4 and 5  Supplementary Materials: Figure 1, 2 and 3 |
|  | 20c | Present results of all investigations of possible causes of heterogeneity among study results. | | Yes  Figure 5 |
|  | 20d | Present results of all sensitivity analyses conducted to assess the robustness of the synthesized results. | | Yes Supplementary Materials: Figure 3 |
| Reporting biases | 21 | Present assessments of risk of bias due to missing results (arising from reporting biases) for each synthesis assessed. | | Yes Supplementary Materials: table 6 |
| Certainty of evidence | 22 | Present assessments of certainty (or confidence) in the body of evidence for each outcome assessed. | | Yes |
| DISCUSSION | | | |  |
| Discussion | 23a | Provide a general interpretation of the results in the context of other evidence. | | Yes |
|  | 23b | Discuss any limitations of the evidence included in the review. | | Yes |
|  | 23c | Discuss any limitations of the review processes used. | | Yes |
|  | 23d | Discuss implications of the results for practice, policy, and future research. | | Yes |
| OTHER INFORMATION | | | |  |
| Registration and protocol | 24a | Provide registration information for the review, including register name and registration number, or state that the review was not registered. | | Yes |
|  | 24b | Indicate where the review protocol can be accessed, or state that a protocol was not prepared. | | Yes |
|  | 24c | Describe and explain any amendments to information provided at registration or in the protocol. | | Yes |
| Support | 25 | Describe sources of financial or non-financial support for the review, and the role of the funders or sponsors in the review. | | Yes |
| Competing interests | 26 | Declare any competing interests of review authors. | | Yes |
| Availability of data, code and other materials | 27 | Report which of the following are publicly available and where they can be found: template data collection forms; data extracted from included studies; data used for all analyses; analytic code; any other materials used in the review. | | Yes |

**Table S2. PRISMA 2020 for Abstract Checklist.**

| Section and Topic | Item # | Checklist item | Reported (Yes/No) |
| --- | --- | --- | --- |
| TITLE | | |  |
| Title | 1 | Identify the report as a systematic review. | Yes |
| BACKGROUND | | |  |
| Objectives | 2 | Provide an explicit statement of the main objective(s) or question(s) the review addresses. | Yes |
| METHODS | | |  |
| Eligibility criteria | 3 | Specify the inclusion and exclusion criteria for the review. | No |
| Information sources | 4 | Specify the information sources (e.g. databases, registers) used to identify studies and the date when each was last searched. | Yes |
| Risk of bias | 5 | Specify the methods used to assess risk of bias in the included studies. | No |
| Synthesis of results | 6 | Specify the methods used to present and synthesise results. | No |
| RESULTS | | |  |
| Included studies | 7 | Give the total number of included studies and participants and summarise relevant characteristics of studies. | Yes |
| Synthesis of results | 8 | Present results for main outcomes, preferably indicating the number of included studies and participants for each. If meta-analysis was done, report the summary estimate and confidence/credible interval. If comparing groups, indicate the direction of the effect (i.e. which group is favoured). | Yes |
| DISCUSSION | | |  |
| Limitations of evidence | 9 | Provide a brief summary of the limitations of the evidence included in the review (e.g. study risk of bias, inconsistency and imprecision). | No |
| Interpretation | 10 | Provide a general interpretation of the results and important implications. | Yes |
| OTHER | | |  |
| Funding | 11 | Specify the primary source of funding for the review. | No |
| Registration | 12 | Provide the register name and registration number. | No |

**Table S3** Inclusion and exclusion criteria of included studies

| **Study** | - **Inclusion Criteria** | **Exclusion Criteria** |
| --- | --- | --- |
| ÇAGLAYAN, 2023 | 1. Patients diagnosed with HR+ (hormone receptor-positive) and HER2-negative metastatic breast cancer (mBC). 2. Patients treated with CDK 4/6 inhibitors (palbociclib or ribociclib). 3. A total of 86 patients were included in the study. 4. Patients divided into two categories based on their concomitant use of proton pump inhibitors (PPIs). | 1. Patients with other types of breast cancer (non-HR+ or HER2-positive). 2. Patients who did not receive CDK 4/6 inhibitors as part of their treatment. 3. Patients with incomplete or unavailable data. 4. Patients using drugs with strong CYP3A4 inhibition. 5. Patients who received CDK 4/6 inhibitors and endocrine therapy as front-line therapy. 6. Patients with a QTc (corrected QT interval) greater than 480 ms. 7. Patients with CDK 4/6 inhibitor-associated pneumonitis. |
| CRIADO, 2023 | 1. Patients diagnosed with metastatic breast cancer (mBC). 2. Patients who received palbociclib for the treatment of mBC between January 2016 and December 2021. 3. Patients treated with hormonal therapy, including fulvestrant or aromatase inhibitors, in combination with palbociclib. 4. Both premenopausal and postmenopausal patients. 5. Patients with different performance statuses. 6. Patients with visceral or non-visceral disease manifestations. 7. Patients in both first-line and second or subsequent lines of treatment. 8. Patients with recorded data on ki67 expression. 9. Patients with or without concomitant use of proton pump inhibitors (PPIs). | 1. Patients who did not receive palbociclib for the treatment of mBC. 2. Patients with incomplete data on the variables of interest. 3. Patients not treated with hormonal therapy in combination with palbociclib. 4. Patients with other types of cancer or medical conditions not related to breast cancer. 5. Patients with contraindications to palbociclib or hormonal therapy. 6. Patients with a history of intolerance or adverse reactions to PPIs. 7. Patients receiving other concurrent medications that may interact with palbociclib. 8. Patients with a history of drug interactions affecting absorption or bioavailability. |
| DEL RE, 2021 | 1. Based on the provided information, here is a list of criteria and characteristics for inclusion and exclusion in this study: 2. Inclusion Criteria: 3. Diagnosis and Treatment:  - Patients diagnosed with metastatic breast cancer (mBC). - Patients who received palbociclib between January 2016 and December 2021. - Patients undergoing hormonal therapy plus palbociclib. - Patients receiving first-line or second-line treatment.  1. Clinical Variables:  - Age (continuous variable). - Menopausal status (premenopausal or postmenopausal). - Performance status (Eastern Cooperative Oncology Group - ECOG). - Hormonal treatment (fulvestrant or aromatase inhibitors). - Presence of visceral or non-visceral disease. - Ki67 expression. - Concomitant use of proton pump inhibitors (PPIs).  1. Outcome Variable:  - Progression-free survival (PFS): Time from starting treatment to progression of the disease. | 1. None explicitly mentioned in the provided text. |
| DEL RE, 2022 | 1. Patients with hormone-positive/HER-2-negative metastatic breast cancer (mBC) candidates for first-line treatment with ribociclib. 2. Patients treated with ribociclib as first-line treatment. 3. Patients classified as "no concomitant PPIs" or "concomitant PPIs." 4. PPI administration covered the entire treatment with ribociclib or not less than 2/3 of it. 5. All clinical interventions were made according to clinical practice. | 1. Patients not meeting the criteria mentioned above.  2. Patients not treated with ribociclib as first-line treatment.  3. Patients not classified as "no concomitant PPIs" or "concomitant PPIs."  4. PPI administration covering less than 2/3 of treatment with ribociclib. |
| ESER, 2022 | 1. Hormone receptor-positive, HER2-negative metastatic breast cancer (mBC) patients. 2. Patients treated with endocrine therapy (letrozole or fulvestrant) combined with palbociclib or ribociclib. 3. Patients treated with "concurrent PPIs" during palbociclib/ribociclib therapy, defined as all or more than half of the treatment duration. 4. Endocrine-sensitive patients (if relapsed at least 12 months after completion of adjuvant endocrine therapy) or endocrine-resistant patients (relapse while receiving adjuvant therapy or recurrence within 12 months of discontinuation of adjuvant endocrine therapy and progression within 6 months after initiating aromatase inhibitor in palliative therapy). 5. Availability of complete data and follow-up of more than 3 months. | 1. Patients using PPIs but less than half of the treatment duration.  2. Patients with a history of chemotherapy in metastatic disease (for some patients).  3. Patients with intolerance to completely discontinue the drug.  4. Patients with less than 3 months of follow-up. |
| LEE, 2023 | 1. Patients with Breast Cancer:   Women diagnosed with advanced or metastatic breast cancer.  Patients with at least 2 claims with code C50 from the International Statistical Classification of Diseases and Related Health Problems, Tenth Revision (ICD-10), during the study period.   1. Palbociclib Treatment:   Patients who received palbociclib between November 1, 2017, and July 31, 2020.  Palbociclib administered continuously for at least 1 cycle (21 days).   1. Concomitant PPI Group:   Patients whose prescriptions for palbociclib and PPI overlapped by at least 33%.  Patients coadministered PPI for more than one-third of the palbociclib treatment duration.   1. Demographic and Clinical Information:   Information on age, menopausal status, treatment combination, Charlson Comorbidity Index score, prior chemotherapy, prior endocrine therapy, and presence of metastases. | 1. Patients using ERBB2-Targeted Drugs:     - Exclusion of patients who used drugs targeting ERBB2 (e.g., trastuzumab, trastuzumab emtansine, pertuzumab, and lapatinib ditosylate) during the study period.  2. Palbociclib Tablet Formulation:     - Limitation to patients using the capsule formulation of palbociclib, as the tablet formulation was approved only in February 2022 in South Korea. |
| ODABAS, 2022 | 1. Patients:   Patients diagnosed with hormone receptor-positive and HER2-negative metastatic breast cancer.  Patients managed with palbociclib or ribociclib as either the initial or subsequent line of therapy.   1. Proton Pump Inhibitors (PPIs):   Patients classified as "concurrent PPIs" if PPIs were given for at least two-thirds of the palbociclib or ribociclib therapy period.   1. Endocrine Sensitivity:   Patients classified as endocrine-sensitive based on the duration of previous endocrine responses (relapse for at least 12 months or de novo advanced breast cancer).   1. Endocrine Resistance:   Patients classified as endocrine-resistant based on relapse after the first 2 years on adjuvant endocrine therapy, relapse within 12 months of completing adjuvant endocrine therapy, or progressive disease ≥ 6 months after initiating endocrine therapy for metastatic breast cancer.   1. Medication Dosage:   Patients using palbociclib 125 mg (capsule form) and ribociclib 600 mg administered once daily for three weeks, followed by 7 days off, repeated every 28 days.   1. 6.Concurrent PPIs Usage:   Patients receiving PPIs such as omeprazole 40 mg, pantoprazole 40 mg, rabeprazole 20 mg, esomeprazole 40 mg, and lansoprazole 30 mg with breakfast.   1. Data Collection Period   Patients included in the study between May 22, 2020, and April 13, 2022. | 1. Patients:     - Patients with HER2-positive breast cancer.     - Patients with a history of using cytochrome P450 3A4 (CYP3A4) antagonists or enhancers.  2. PPI Dosage:     - Patients not receiving PPIs in the specified dosages.  3. Gastric pH Changes:     - Patients with changes in gastric pH not primarily due to the use of proton pump inhibitors.  4. Medication Dosage Adjustments:     - Patients with dosage adjustments other than those specified for palbociclib and ribociclib based on tolerability.  5. Concomitant Medications:     - Patients using medications that may significantly interact with palbociclib or ribociclib.  6. CYP3A4 Metabolism:     - Patients using medications affecting CYP3A4 metabolism.  7. PPI Usage:     - Patients not using PPIs as specified for inclusion criteria.  8. Breakfast Administration:     - Patients not taking PPIs with breakfast as specified. |
| PARSIVAL, 2023 | 1. Patient Population:   Patients with hormone receptor-positive, HER2-negative advanced breast cancer (HR+/HER2-ABC).   1. Treatment History:   Patients with no prior therapy in the advanced setting.   1. Treatment Assignment:   Patients randomly assigned to receive Palbociclib (PAL) plus either fulvestrant or letrozole.   1. Proton Pump Inhibitor (PPI) Use:   Patients with at least one PPI received over the entire PAL-based regimen.   1. Subgroups for Analysis:   Early PPI users (E-PPI): Patients receiving PPI since the PAL-based regimen initiation.  Long-term PPI users (LT-PPI): Patients who received PPI over the entire or ≥2⁄3 of the PAL-based regimen. | 1. PPI Limited Exposure:     - PPI users defined as neither E-PPI nor LT-PPI were excluded from the analysis to avoid biases due to limited PPI exposure. |
| SCHIEBER, 2023 | 1. Patients diagnosed with hormone receptor-positive (HR+) human epidermal growth factor receptor 2 negative (HER2-) metastatic breast cancer (MBC). 2. Patients treated with palbociclib tablets in the first-line setting. 3. Patients with or without a proton pump inhibitor (PPI) during palbociclib therapy. 4. Adult pre-menopausal women on tamoxifen monotherapy, as well as post-menopausal women and men. 5. Patients with bone-only metastatic disease or other metastatic patterns. 6. Patients with ER levels >1% and PR levels >1%, with a majority having ER levels >50%. 7. Patients who received a concurrent PPI at initiation and continued for >50% of the treatment duration. 8. Patients with palliative radiation use and/or prior chemotherapy. | 1. Patients with less than 50% duration of concurrent PPI use during palbociclib therapy.  2. Patients diagnosed with a malabsorption condition.  3. Patients receiving a single dose of palbociclib capsules (excluding those who received palbociclib tablets).  4. Patients with ER/PR levels <10%.  5. Patients with intolerance or contraindication to an aromatase inhibitor using tamoxifen monotherapy as the endocrine backbone.  6. Patients receiving palbociclib capsules. |

**Table S4** Search Strategies

| **Database** | **Search Strategy** |
| --- | --- |
| **PubMed** | ((((((((((((((((((((((((((((((((((((("breast neoplasms"[MeSH Terms]) OR ("breast neoplasms"[Title/Abstract])) OR ("neoplasm, breast"[Title/Abstract])) OR ("breast tumors"[Title/Abstract])) OR ("breast tumor"[Title/Abstract])) OR ("tumor, breast"[Title/Abstract])) OR ("tumors, breast"[Title/Abstract])) OR ("neoplasms, breast"[Title/Abstract])) OR ("breast cancer"[Title/Abstract])) OR ("cancer, breast"[Title/Abstract])) OR ("mammary cancer"[Title/Abstract])) OR ("cancer, mammary"[Title/Abstract])) OR ("cancers, mammary"[Title/Abstract])) OR ("mammary cancers"[Title/Abstract])) OR ("malignant neoplasm of breast"[Title/Abstract])) OR ("breast malignant neoplasm"[Title/Abstract])) OR ("breast malignant neoplasms"[Title/Abstract])) OR ("malignant tumor of breast"[Title/Abstract])) OR ("breast malignant tumor"[Title/Abstract])) OR ("breast malignant tumors"[Title/Abstract])) OR ("cancer of breast"[Title/Abstract])) OR ("cancer of the breast"[Title/Abstract])) OR ("mammary carcinoma, human"[Title/Abstract])) OR ("carcinoma, human mammary"[Title/Abstract])) OR ("carcinomas, human mammary"[Title/Abstract])) OR ("human mammary carcinomas"[Title/Abstract])) OR ("mammary carcinomas, human"[Title/Abstract])) OR ("human mammary carcinoma"[Title/Abstract])) OR ("mammary neoplasms, human"[Title/Abstract])) OR ("human mammary neoplasm"[Title/Abstract])) OR ("human mammary neoplasms"[Title/Abstract])) OR ("neoplasm, human mammary"[Title/Abstract])) OR ("neoplasms, human mammary"[Title/Abstract])) OR ("mammary neoplasm, human"[Title/Abstract])) OR ("breast carcinoma"[Title/Abstract])) OR ("breast carcinomas"[Title/Abstract])) OR ("carcinoma, breast"[Title/Abstract])) OR ("carcinomas, breast"[Title/Abstract]) AND ((((((((((((((((((((((((((((((((((((((((((((((((((((((((((((((("Cyclin-Dependent Kinase Inhibitor Proteins"[MeSH Terms]) OR ("Cyclin-Dependent Kinase Inhibitor Proteins"[Title/Abstract])) OR ("Cyclin Dependent Kinase Inhibitor Proteins"[Title/Abstract])) OR ("CKI Proteins"[Title/Abstract])) OR ("CDKI Proteins"[Title/Abstract])) OR ("Cyclin-Kinase Inhibitor Proteins"[Title/Abstract])) OR ("Cyclin Kinase Inhibitor Proteins"[Title/Abstract])) OR ("Inhibitor Proteins, Cyclin-Kinase"[Title/Abstract])) OR ("CIP-KIP Cyclin-Dependent Kinase Inhibitors"[Title/Abstract])) OR ("CIP KIP Cyclin Dependent Kinase Inhibitors"[Title/Abstract])) OR ("CIP-KIP CKI Proteins"[Title/Abstract])) OR ("CIP KIP CKI Proteins"[Title/Abstract])) OR ("CKI Proteins, CIP- KIP"[Title/Abstract])) OR ("CIP-KIP CDKI Proteins"[Title/Abstract])) OR ("CDKI Proteins, CIP-KIP"[Title/Abstract])) OR ("CIP KIP CDKI Proteins"[Title/Abstract])) OR ("INK4 Cyclin-Dependent Kinase Inhibitors"[Title/Abstract])) OR ("INK4 Cyclin Dependent Kinase Inhibitors"[Title/Abstract])) OR ("INK4 CDKI Proteins"[Title/Abstract])) OR ("CDKI Proteins, INK4"[Title/Abstract])) OR ("INK4 CKI Proteins"[Title/Abstract])) OR ("CKI Proteins, INK4"[Title/Abstract])) OR ("Inhibitors of Cyclin- Dependent Kinase 4 Proteins"[Title/Abstract])) OR ("Inhibitors of Cyclin Dependent Kinase 4 Proteins"[Title/Abstract])) OR ("Protein Kinase Inhibitors"[Title/Abstract])) OR ("Inhibitors, Protein Kinase"[Title/Abstract])) OR ("Kinase Inhibitors, Protein"[Title/Abstract])) OR ("Protein Kinase Inhibitor"[Title/Abstract])) OR ("Inhibitor, Protein Kinase"[Title/Abstract])) OR ("Kinase Inhibitor, Protein"[Title/Abstract])) OR ("CDK-4"[Title/Abstract] OR "CDK 4"[Title/Abstract])) OR ("cyclin-dependent kinase 4"[Title/Abstract])) OR ("cyclin dependent kinase 4"[Title/Abstract])) OR ("CDK-6"[Title/Abstract] OR "CDK 6"[Title/Abstract])) OR ("cyclin- dependent kinase 6"[Title/Abstract])) OR ("cyclin dependent kinase 6"[Title/Abstract])) OR ("Protein Kinase Inibitors"[Title/Abstract])) OR ("Abemaciclib"[Title/Abstract])) OR ("Palbociclib"[Title/Abstract])) OR ("Ribociclib"[Title/Abstract])) OR ("Cyclin-Dependent Kinase 4"[Title/Abstract])) OR ("Cyclin Dependent Kinase 4"[Title/Abstract])) OR ("Cdk4 Cyclin-Dependent Kinase"[Title/Abstract])) OR ("Cdk4 Cyclin Dependent Kinase"[Title/Abstract])) OR ("Cyclin-Dependent Kinase, Cdk4"[Title/Abstract])) OR (Cyclin-Dependent Kinase 4[MeSH Terms])) OR ("Cdk4 Protein"[Title/Abstract])) OR ("Cdk4 Protein Kinase"[Title/Abstract])) OR ("Protein Kinase, Cdk4"[Title/Abstract])) OR ("p34PSK-J3 Kinase"[Title/Abstract])) OR ("p34PSK J3 Kinase"[Title/Abstract])) OR ("Cell Division Protein Kinase 4"[Title/Abstract])) OR ("PSK-J3 Kinase"[Title/Abstract])) OR ("PSK J3 Kinase"[Title/Abstract])) OR ("Cyclin D- Dependent Kinase CDK4"[Title/Abstract])) OR ("Cyclin D Dependent Kinase CDK4"[Title/Abstract])) OR ("Cyclin-Dependent Kinase 6"[Title/Abstract])) OR ("Cyclin Dependent Kinase 6"[Title/Abstract])) OR ("Cyclin-Dependent Kinase 6"[MeSH Terms])) OR ("Cell Division Protein Kinase 6"[Title/Abstract])) OR ("Cdk6 Protein Kinase"[Title/Abstract])) OR ("Protein Kinase, Cdk6"[Title/Abstract])) OR ("CDK6 Protein"[Title/Abstract])) OR ("PLSTIRE Protein"[Title/Abstract]) AND ((((((((((((((((("Proton Pump Inhibitors"[MeSH Terms]) OR ("Proton Pump Inhibitors"[Title/Abstract])) OR ("Inhibitors, Proton Pump"[Title/Abstract])) OR ("Proton Pump Inhibitor"[Title/Abstract])) OR ("Inhibitor, Proton Pump"[Title/Abstract])) OR ("Pump Inhibitor, Proton"[Title/Abstract])) OR ("Omeprazole"[MeSH Terms])) OR ("Omeprazole"[Title/Abstract])) OR ("Lansoprazole"[MeSH Terms])) OR ("Lansoprazole"[Title/Abstract])) OR (Esomeprazole[MeSH Terms])) OR ("Esomeprazol"[Title/Abstract])) OR ("Pantoprazole"[MeSH Terms])) OR ("Pantoprazole"[Title/Abstract])) OR ("Rabeprazole"[MeSH Terms])) OR ("Rabeprazole"[Title/Abstract])) OR ("Dexlansoprazole"[MeSH Terms])) OR ("Dexlansoprazole"[Title/Abstract]) |
| **Web of Science** | (((((((((((((((((((((((((((((((((((((TS=("breast neoplasm")) OR TS=("neoplasm, breast")) OR TS=("breast tumors")) OR TS=("breast tumor")) OR TS=("tumor, breast")) OR TS=("tumors, breast")) OR TS=("neoplasms, breast")) OR TS=("breast cancer")) OR TS=("cancer, breast")) OR TS=("mammary cancer")) OR TS=("cancer, mammary")) OR TS=("cancers, mammary")) OR TS=("mammary cancers")) OR TS=("malignant neoplasm of breast")) OR TS=("breast malignant neoplasm")) OR TS=("breast malignant neoplasms")) OR TS=("malignant tumor of breast")) OR TS=("breast malignant tumor")) OR TS=("breast malignant tumors")) OR TS=("cancer of breast")) OR TS=("cancer of the breast")) OR TS=("mammary carcinoma, human")) OR TS=("carcinoma, human mammary")) OR TS=("carcinomas, human mammary")) OR TS=("human mammary carcinomas")) OR TS=("mammary carcinomas, human")) OR TS=("human mammary carcinoma")) OR TS=("mammary neoplasms, human")) OR TS=("human mammary neoplasm")) OR TS=("human mammary neoplasms")) OR TS=("neoplasm, human mammary")) OR TS=("neoplasms, human mammary")) OR TS=("mammary neoplasm, human")) OR TS=("breast carcinoma")) OR TS=("breast carcinomas")) OR TS=("carcinoma, breast")) OR TS=("carcinomas, breast")) AND (((((((((((((((((((((((((((((((((((((((((((((((((((((((((((((((TS=(“Cyclin-Dependent Kinase Inhibitor Proteins”)) OR TS=(“Cyclin Dependent Kinase Inhibitor Proteins”)) OR TS=(“CKI Proteins”)) OR TS=(“CDKI Proteins”)) OR TS=(“Cyclin-Kinase Inhibitor Proteins”)) OR TS=(“Cyclin Kinase Inhibitor Proteins”)) OR TS=(“Inhibitor Proteins, Cyclin-Kinase”)) OR TS=(“CIP-KIP Cyclin-Dependent Kinase Inhibitors”)) OR TS=(“CIP KIP Cyclin Dependent Kinase Inhibitors”)) OR TS=(“CIP-KIP CKI Proteins”)) OR TS=(“CIP KIP CKI Proteins”)) OR TS=(“CKI Proteins, CIP- KIP”)) OR TS=(“CIP-KIP CDKI Proteins”)) OR TS=(“CDKI Proteins, CIP-KIP”)) OR TS=(“CDKI Proteins, CIP-KIP”)) OR TS=(“INK4 Cyclin-Dependent Kinase Inhibitors”)) OR TS=(“INK4 Cyclin Dependent Kinase Inhibitors”)) OR TS=(“INK4 CDKI Proteins”)) OR TS=(“CDKI Proteins, INK4”)) OR TS=(“INK4 CKI Proteins”)) OR TS=(“CKI Proteins, INK4”)) OR TS=(“Inhibitors of Cyclin- Dependent Kinase 4 Proteins”)) OR TS=(“Inhibitors of Cyclin Dependent Kinase 4 Proteins”)) OR TS=(“Protein Kinase Inhibitors”)) OR TS=(“Inhibitors, Protein Kinase”)) OR TS=(“Kinase Inhibitors, Protein”)) OR TS=(“Protein Kinase Inhibitor”)) OR TS=(“Inhibitor, Protein Kinase”)) OR TS=(“Kinase Inhibitor, Protein”)) OR TS=("CDK-4")) OR TS=("CDK 4")) OR TS=("cyclin-dependent kinase 4")) OR TS=("cyclin dependent kinase 4")) OR TS=("CDK-6")) OR TS=("CDK 6")) OR TS=("cyclin- dependent kinase 6")) OR TS=("cyclin dependent kinase 6")) OR TS=(“Protein Kinase Inibitors")) OR TS=(“Abemaciclib”)) OR TS=(“Palbociclib”)) OR TS=(“Ribociclib")) OR TS=(“Cyclin-Dependent Kinase 4")) OR TS=(“Cyclin Dependent Kinase 4”)) OR TS=(“Cdk4 Cyclin-Dependent Kinase”)) OR TS=(“Cdk4 Cyclin Dependent Kinase”)) OR TS=(“Cyclin-Dependent Kinase, Cdk4”)) OR TS=(“Cdk4 Protein”)) OR TS=(“Cdk4 Protein Kinase”)) OR TS=(“Protein Kinase, Cdk4”)) OR TS=(“p34PSK-J3 Kinase”)) OR TS=(“p34PSK J3 Kinase”)) OR TS=(“Cell Division Protein Kinase 4”)) OR TS=(“PSK-J3 Kinase”)) OR TS=(“PSK J3 Kinase”)) OR TS=(“Cyclin D- Dependent Kinase CDK4”)) OR TS=(“Cyclin D Dependent Kinase CDK4”)) OR TS=(“Cyclin-Dependent Kinase 6”)) OR TS=(“Cyclin Dependent Kinase 6”)) OR TS=(“Cell Division Protein Kinase 6”)) OR TS=(“Cdk6 Protein Kinase”)) OR TS=(“Protein Kinase, Cdk6”)) OR TS=(“CDK6 Protein”)) OR TS=(“PLSTIRE Protein”)) AND (((((((((((TS=(“Proton Pump Inhibitors”)) OR TS=("Inhibitors, Proton Pump")) OR TS=("Proton Pump Inhibitor")) OR TS=("Inhibitor, Proton Pump")) OR TS=("Pump Inhibitor, Proton")) OR TS=("Omeprazole")) OR TS=("Lansoprazole")) OR TS=("Esomeprazole")) OR TS=("Pantoprazole")) OR TS=("Rabeprazole")) OR TS=("Dexlansoprazole")) |
| **Embase** | ( TITLE-ABS-KEY ( "breast neoplasm" ) OR TITLE-ABS-KEY ( "neoplasm, breast" ) OR TITLE-ABS-KEY ( "breast tumors" ) OR TITLE-ABS-KEY ( "breast tumor" ) OR TITLE-ABS-KEY ( "tumor, breast" ) OR TITLE-ABS-KEY ( "tumors, breast" ) OR TITLE-ABS-KEY ( "neoplasms, breast" ) OR TITLE-ABS-KEY ( "breast cancer" ) OR TITLE-ABS-KEY ( "cancer, breast" ) OR TITLE-ABS-KEY ( "mammary cancer" ) OR TITLE-ABS-KEY ( "cancer, mammary" ) OR TITLE-ABS-KEY ( "cancers, mammary" ) OR TITLE-ABS-KEY ( "mammary cancers" ) OR TITLE-ABS-KEY ( "malignant neoplasm of breast" ) OR TITLE-ABS-KEY ( "breast malignant neoplasm" ) OR TITLE-ABS-KEY ( "breast malignant neoplasms" ) OR TITLE-ABS-KEY ( "malignant tumor of breast" ) OR TITLE-ABS-KEY ( "breast malignant tumor" ) OR TITLE-ABS-KEY ( "breast malignant tumors" ) OR TITLE-ABS-KEY ( "cancer of breast" ) OR TITLE-ABS-KEY ( "cancer of the breast" ) OR TITLE-ABS-KEY ( "mammary carcinoma, human" ) OR TITLE-ABS-KEY ( "carcinoma, human mammary" ) OR TITLE-ABS-KEY ( "carcinomas, human mammary" ) OR TITLE-ABS-KEY ( "human mammary carcinomas" ) OR TITLE-ABS-KEY ( "mammary carcinomas, human" ) OR TITLE-ABS-KEY ( "human mammary carcinoma" ) OR TITLE-ABS-KEY ( "mammary neoplasms, human" ) OR TITLE-ABS-KEY ( "human mammary neoplasm" ) OR TITLE-ABS-KEY ( "human mammary neoplasms" ) OR TITLE-ABS-KEY ( "neoplasm, human mammary" ) OR TITLE-ABS-KEY ( "neoplasms, human mammary" ) OR TITLE-ABS-KEY ( "mammary neoplasm, human" ) OR TITLE-ABS-KEY ( "breast carcinoma" ) OR TITLE-ABS-KEY ( "breast carcinomas" ) OR TITLE-ABS-KEY ( "carcinoma, breast" ) OR TITLE-ABS-KEY ( "carcinomas, breast" ) ) AND ( TITLE-ABS-KEY ( "Cyclin-Dependent Kinase Inhibitor Proteins" ) OR TITLE-ABS-KEY ( "Cyclin Dependent Kinase Inhibitor Proteins" ) OR TITLE-ABS-KEY ( "CKI Proteins" ) OR TITLE-ABS-KEY ( "CDKI Proteins" ) OR TITLE-ABS-KEY ( "Cyclin-Kinase Inhibitor Proteins" ) OR TITLE-ABS-KEY ( "Cyclin Kinase Inhibitor Proteins" ) OR TITLE-ABS-KEY ( "Inhibitor Proteins, Cyclin-Kinase" ) OR TITLE-ABS-KEY ( "CIP-KIP Cyclin-Dependent Kinase Inhibitors" ) OR TITLE-ABS-KEY ( "CIP KIP Cyclin Dependent Kinase Inhibitors" ) OR TITLE-ABS-KEY ( "CIP-KIP CKI Proteins" ) OR TITLE-ABS-KEY ( "CIP KIP CKI Proteins" ) OR TITLE-ABS-KEY ( "CKI Proteins, CIP- KIP" ) OR TITLE-ABS-KEY ( "CIP-KIP CDKI Proteins" ) OR TITLE-ABS-KEY ( "CDKI Proteins, CIP-KIP" ) OR TITLE-ABS-KEY ( "CIP KIP CDKI Proteins" ) OR TITLE-ABS-KEY ( "INK4 Cyclin-Dependent Kinase Inhibitors" ) OR TITLE-ABS-KEY ( "INK4 Cyclin Dependent Kinase Inhibitors" ) OR TITLE-ABS-KEY ( "INK4 CDKI Proteins" ) OR TITLE-ABS-KEY ( "CDKI Proteins, INK4" ) OR TITLE-ABS-KEY ( "INK4 CKI Proteins" ) OR TITLE-ABS-KEY ( "CKI Proteins, INK4" ) OR TITLE-ABS-KEY ( "Inhibitors of Cyclin- Dependent Kinase 4 Proteins" ) OR TITLE-ABS-KEY ( "Inhibitors of Cyclin Dependent Kinase 4 Proteins" ) OR TITLE-ABS-KEY ( "Protein Kinase Inhibitors" ) OR TITLE-ABS-KEY ( "Inhibitors, Protein Kinase" ) OR TITLE-ABS-KEY ( "Kinase Inhibitors, Protein" ) OR TITLE-ABS-KEY ( "Protein Kinase Inhibitor" ) OR TITLE-ABS-KEY ( "Inhibitor, Protein Kinase" ) OR TITLE-ABS-KEY ( "Kinase Inhibitor, Protein" ) OR TITLE-ABS-KEY ( "CDK-4" ) OR TITLE-ABS-KEY ( "CDK 4" ) OR TITLE-ABS-KEY ( "cyclin-dependent kinase 4" ) OR TITLE-ABS-KEY ( "cyclin dependent kinase 4" ) OR TITLE-ABS-KEY ( "CDK-6" ) OR TITLE-ABS-KEY ( "CDK 6" ) OR TITLE-ABS-KEY ( "cyclin-dependent kinase 6" ) OR TITLE-ABS-KEY ( "cyclin dependent kinase 6" ) OR TITLE-ABS-KEY ( "Protein Kinase Inibitors" ) OR TITLE-ABS-KEY ( "Abemaciclib" ) OR TITLE-ABS-KEY ( "Palbociclib" ) OR TITLE-ABS-KEY ( "Ribociclib" ) OR TITLE-ABS-KEY ( "Cyclin-Dependent Kinase 4" ) OR TITLE-ABS-KEY ( "Cyclin Dependent Kinase 4" ) OR TITLE-ABS-KEY ( "Cdk4 Cyclin-Dependent Kinase" ) OR TITLE-ABS-KEY ( "Cdk4 Cyclin Dependent Kinase" ) OR TITLE-ABS-KEY ( "Cyclin-Dependent Kinase, Cdk4" ) OR TITLE-ABS-KEY ( "Cdk4 Protein" ) OR TITLE-ABS-KEY ( "Cdk4 Protein Kinase" ) OR TITLE-ABS-KEY ( "Protein Kinase, Cdk4" ) OR TITLE-ABS-KEY ( "p34PSK-J3 Kinase" ) OR TITLE-ABS-KEY ( "p34PSK J3 Kinase" ) OR TITLE-ABS-KEY ( "Cell Division Protein Kinase 4" ) OR TITLE-ABS-KEY ( "PSK-J3 Kinase" ) OR TITLE-ABS-KEY ( "PSK J3 Kinase" ) OR TITLE-ABS-KEY ( "Cyclin D-Dependent Kinase CDK4" ) OR TITLE-ABS-KEY ( "Cyclin D Dependent Kinase CDK4" ) OR TITLE-ABS-KEY ( "Cyclin-Dependent Kinase 6" ) OR TITLE-ABS-KEY ( "Cyclin Dependent Kinase 6" ) OR TITLE-ABS-KEY ( "Cell Division Protein Kinase 6" ) OR TITLE-ABS-KEY ( "Cdk6 Protein Kinase" ) OR TITLE-ABS-KEY ( "Protein Kinase, Cdk6" ) OR TITLE-ABS-KEY ( "CDK6 Protein" ) OR TITLE-ABS-KEY ( "PLSTIRE Protein" ) ) AND ( TITLE-ABS-KEY ( "Proton Pump Inhibitors" ) OR TITLE-ABS-KEY ( "Inhibitors, Proton Pump" ) OR TITLE-ABS-KEY ( "Proton Pump Inhibitor" ) OR TITLE-ABS-KEY ( "Inhibitor, Proton Pump" ) OR TITLE-ABS-KEY ( "Pump Inhibitor, Proton" ) OR TITLE-ABS-KEY ( "Omeprazole" ) OR TITLE-ABS-KEY ( "Lansoprazole" ) OR TITLE-ABS-KEY ( "Esomeprazole" ) OR TITLE-ABS-KEY ( "Pantoprazole" ) OR TITLE-ABS-KEY ( "Rabeprazole" ) OR TITLE-ABS-KEY ( "Dexlansoprazole" ) ) |

**Table S5** Treatment regimens from included studies in this systematic review and meta-analysis

| **Study** | **Treatment regimes** |
| --- | --- |
| Çaglayan, 2023 | Pacientes were divided into two groups: those who used proton pump inhibitors (PPIs) concurrently with CDK 4/6 inhibitors (ribociclib or palpociclib) and those who did not. The proportion of patients in each group wasn`t explicitly mentioned in the provided context. However, it was stated that 45 (52.3%) patients used concomitant PPIs with CDK 4/6 inhibitors, while 41 (47.7%) did not. The study doesn`t provide specific information about the doses of medications used and the duration of treatment regimens. The PPIs used were rabeprazole (20mg), esomeprazole (40mg), pantoprazole (40mg), and lansoprazole (30mg). The endocrine therapy (fulvestrant or aromatase inhibitors) was used too. |
| Criado, 2023 | Patients with metastatic breast cancer treated with hormonal therapy (fulvestrant or aromatase inhibitors) plus Palbociclib who 80 patients receive PPI and 86 did not. Duration of Palbociclib treatment wasn`t explicitly mentioned. |
| Del Re, 2021 | The treatment regimen consisted of Palbociclib, an oral CDK 4.6 inhibitor, in combination with endocrine therapy. Patients were divided into two groups: one group consisted of 56 patients who did not reveive any PPIs during palbociblib treatment, and the other group consisted of 56 patients who received concomitant PPIs along with Palbociclib treatment. The specific PPIs used were lansoprazole (15mg), esomeprazole (20mg), omeprazole (10mg), or pantoprazole (20mg), taken in the morning at breakfast. Palbociblib was taken at lunchtime. The doses of Palbociclib varied, with the majority of patients receiving 125mg (61.6%), followed by a reduction to 100mg (26.8%) and a small percentage needing a dose of 75mg (9.8%). The patients were followed up to assess progression-free survival (PFS), which is defined as the time from treatment start to disease progression. The follow-up period and specific duration weren`t mentioned. The study included a total of 112 patients with metastatic breast cancer (mBC). These patients were divided into two groups: one group consisted of 56 patients who did not receive any PPIs during Palbociclib treatment, and the other group consisted of 56 patients who received concomitant PPIs along with palpociclib treatment. |
| Del Re, 2022 | This retrospective-cohort study based on reviewing medical charts of HR-positive/HER2-negative metastatic breast cancer patients treated with ribociclib. Included a total of 128 participants divided into two groups: concomitant PPIs (50 patients) and no concomitant PPIs (78 patients). The treatment regimen involved the administration of ribociclib orally at a dose of 600mg once daily for 21 days on/7 days off in 28-day cycles, along with endocrine therapy (fulvestrant or letrozole) according to clinical practice. Participants were stratified based on endocrine sensitivity or resistance. |
| Eser, 2022 | This retrospective observational study involved patients who were already receiving treatment with either palbociclib or ribociclib for hormone receptor-positive, HER2-negative metastatic breast cancer. Included a total of 217 patients, with 105 patients receiving palbociclib and 112 patients receiving ribociclib. Participants were divided based on their use of proton pump inhibitors (PPIs). In the palbociclib group, 65 patients were on concomitant PPI therapy, while 40 were not. In the ribociclib group, 61 patients were on concomitant PPI therapy, while 51 were not. The treatment regimen involved the combination of palbociclib or ribociclib with either letrozole or fulvestrant as endocrine therapy. Dose reductions of the CDK inhibitors were performed based on the toxicity profile. For palbociclib, the oral dose was 125 mg, and for ribociclib, the oral dose was 600 mg/21 days on and 7 days off. Ribociclib dose reduction was made to 400 mg, and palbociclib dose reduction was made to 100 mg. It's important to note that no lower dose was used in any patient. The PPIs used included lansoprazole, esomeprazole, omeprazole, pantoprazole, and rabeprazole. |
| Lee, 2023 | This retrospective cohort study identified 1,310 patients with breast cancer who received palbociclib between November 2017 and July 2020. Participants were divided into two groups: those who concomitantly used proton pump inhibitors (PPIs) with palbociclib and those who did not use PPIs during palbociclib treatment. The proportion of participants in each group was not specified. Involved the use of palbociclib, a cyclin-dependent kinase 4/6 inhibitor, in combination with nonsteroidal aromatase inhibitors such as anastrozole or letrozole in most patients. |
| Odabas, 2022 | This retrospective observational study of patients with hormone receptor-positive and HER2-negative metastatic breast cancer who were treated with either palbociclib or ribociclib. included a total of 220 patients, with 120 receiving palbociclib and 100 receiving ribociclib. Participants were divided into two groups based on whether they received concurrent proton pump inhibitors (PPIs) or not. In the palbociclib group, 63 patients received PPIs while 57 did not. In the ribociclib group, 29 patients received PPIs while 71 did not. Involved the use of palbociclib 125 mg or ribociclib 600 mg once daily for three weeks, followed by a 7-day break, and this cycle was repeated every 28 days. The dose of medications could be adjusted based on tolerability. |
| Parsival, 2023 | This randomized phase 2 open-label clinical trial with two parallel groups. The population was randomized in a 1:1 ratio to receive either fulvestrant plus palbociclib or letrozole plus palbociclib. The study enrolled a total of 486 women, with 243 assigned to each treatment group. The treatment regimen consisted of either fulvestrant plus palbociclib or letrozole plus palbociclib. The participants were divided based on their menopausal status, type of disease (de novo or recurrent), and the presence or absence of visceral involvement. The dose intensity of the medications varied, with the median relative dose intensity being 99.2% for fulvestrant and 91.7% for palbociclib in the fulvestrant-palbociclib group, and 98.8% for letrozole and 90.0% for palbociclib in the letrozole-palbociclib group.  Among them, 66.9% (325 patients) were not proton pump inhibitor (PPI) users (N-PPI), while 33.1% (161 patients) were PPI users. The PPI users were further categorized into early PPI users (E-PPI) and long-term PPI users (LT-PPI) based on the duration of PPI use.  The most prescribed PPI among the PPI users was omeprazole, which was used by 80.7% of the PPI users. The median duration of PPI exposure for PPI users, E-PPI, and LT-PPI was 13.6, 15.9, and 19.4 months, respectively. |
| Schieber, 2023 | This retrospective observational study. participants were included a total of 82 patients, with 50 patients in the no PPI use arm and 32 patients in the PPI use arm. The treatment regimen involved the use of palbociclib tablets with or without a proton pump inhibitor (PPI). Participants were divided into two groups based on whether they received a PPI or not. The dose of medications was not specified. |

**Supplementary Table 6** Quality assessment using the Newcastle-Ottawa Scale (NOS) of include studies in systematic review and meta-analysis.

| **Meta-data** | | **Methodology** | **Newcastle-Ottawa Scale** | | | |
| --- | --- | --- | --- | --- | --- | --- |
| **Author** | **Publication Date** | ***Study design**** | ***Selection*** | ***Comparability*** | ***Exposure*** | ***Quality*** |
| Çaglayan | *2023* | *-+* | ☆☆☆☆ | ☆☆ | ☆☆☆ | 9 |
| Criado | *2023* | *-* | ☆☆☆ | ☆☆ | ☆☆☆ | 8 |
| Del Re | *2021* | *-+* | ☆☆☆☆ | ☆☆ | ☆☆☆ | 9 |
| Del Re | *2022* | *-+* | ☆☆☆☆ | ☆☆ | ☆☆☆ | 9 |
| Eser | *2022* | *-+* | ☆☆☆☆ | ☆☆ | ☆ | 7 |
| Lee | *2023* | *--* | ☆☆☆☆ | ☆ | ☆☆ | 7 |
| Odabas | *2022* | *--* | ☆☆☆☆ | ☆☆ | ☆☆☆ | 9 |
| Parsival | *2023* | *++* | ☆☆☆ | ☆ | ☆☆ | 6 |
| Schieber | *2023* | *--* | ☆☆☆☆ | ☆☆ | ☆☆☆ | 9 |

*Abbreviations*: *Study design: Prospective (+), Retrospective (−); single centre (−), multicentre (+) , Maximum quality score = 9; 0–7 points were considered lower quality, and 8–9 points were considered as higher quality

**Supplementary Figure 1** Univariate Analysis: A- Dose Reduction; B- Metastatic sites; C – Metastasis Visceral or Non-visceral; D- Pre or Post-menopause

A – Dose Reduction


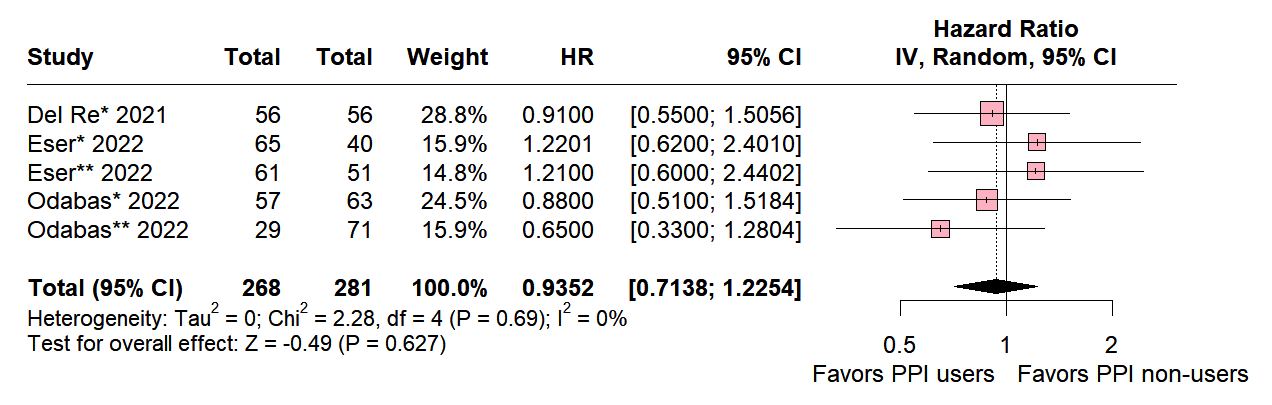


B – Metastatic Sites


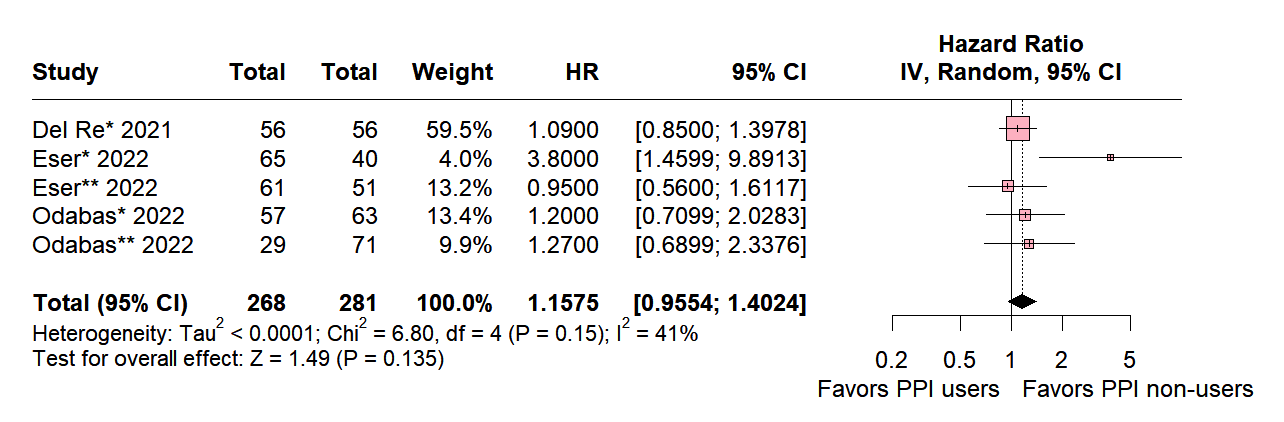


C – Metastasis Visceral or Non-visceral


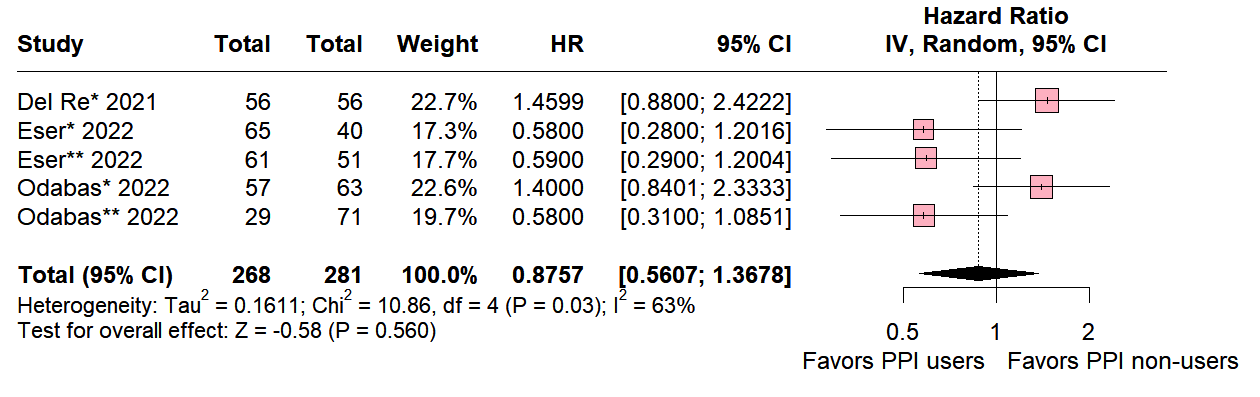


D – Pre or Post-menopause

**
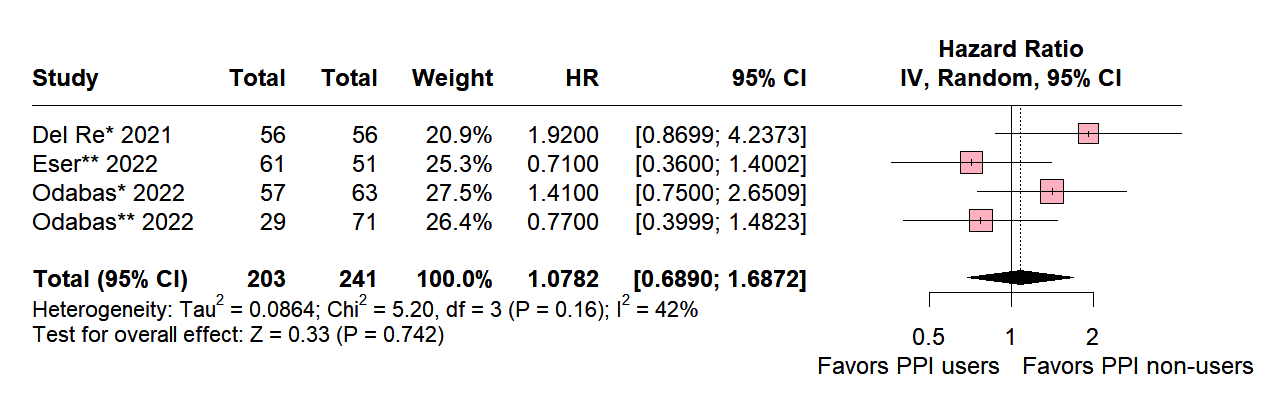
**

**Supplementary Figure 2** Multivariate Analysis: A- ECOG; B – Age

1. ECOG


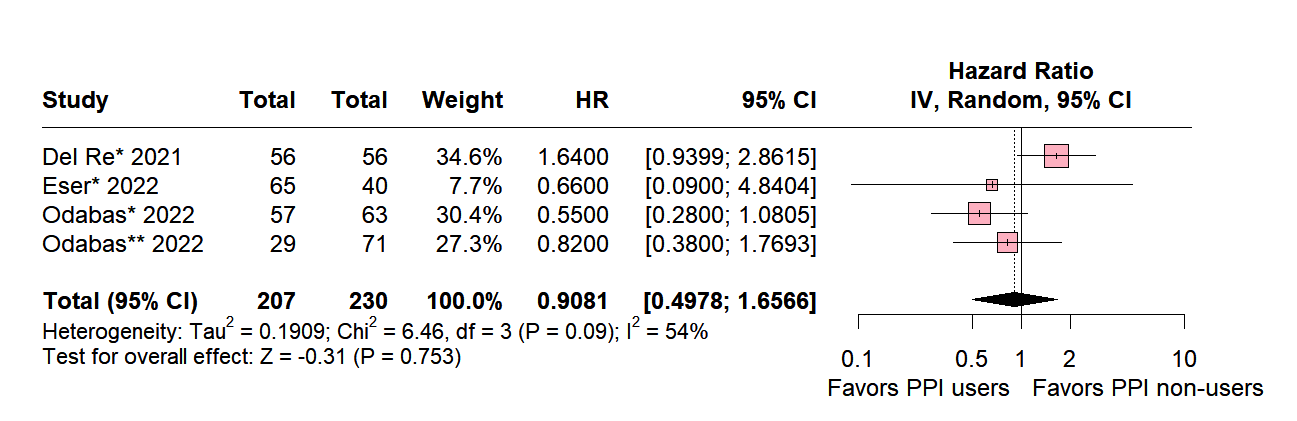


1. Age


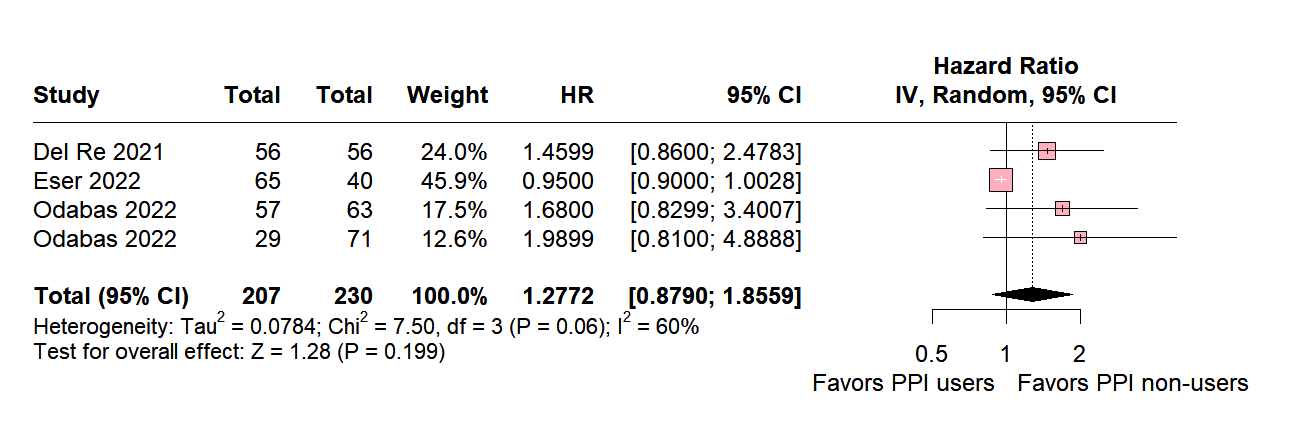


**Supplementary Figure** A- Sensitive PFS Overall; B- Sensitive PFS Palbo; C- Sensitive PFS Ribo; D- Baujart overall PFS; E- Funnel Plot Palbo; F- Funnel Plot Ribo PFS; G- Baujart Ribo PFS

1. Sensitive PFS Overall


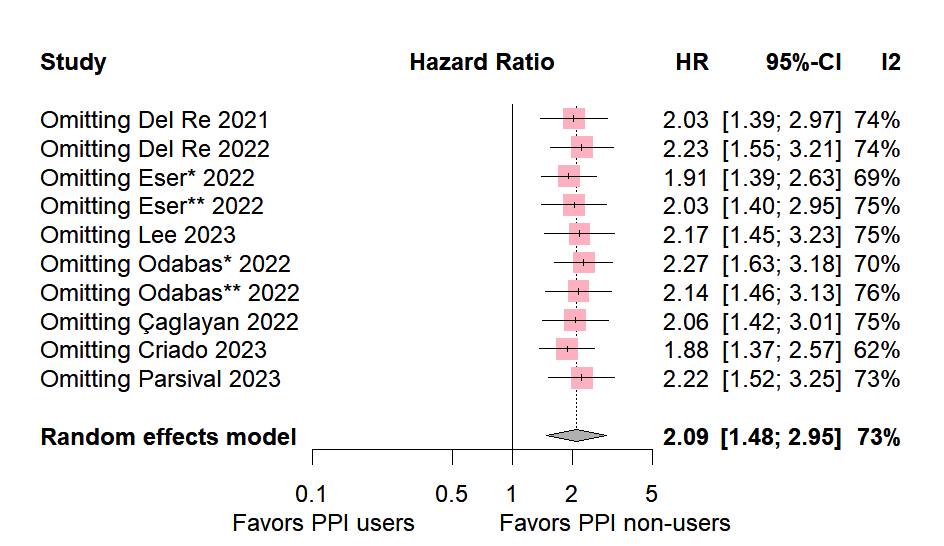


1. Sensitive PFS Palbo


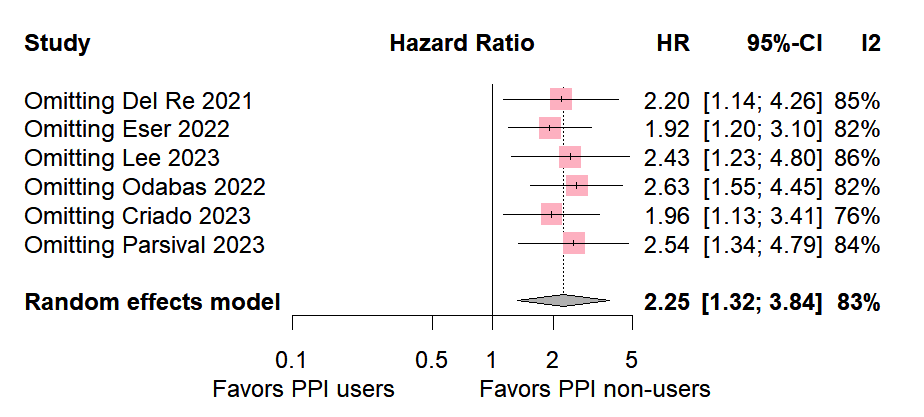


1. Sensitive PFS Ribo


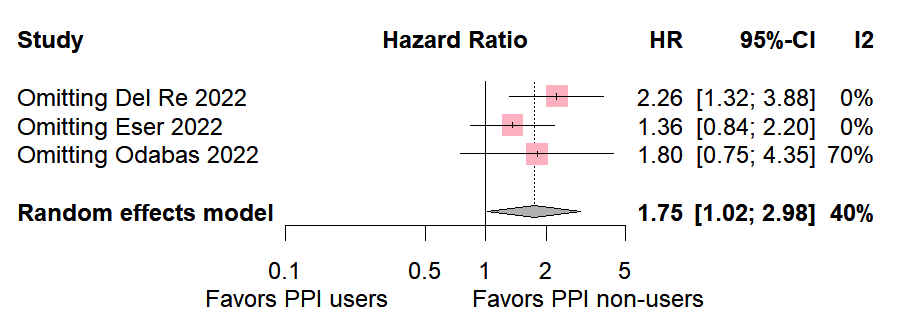


1. Baujart overall PFS


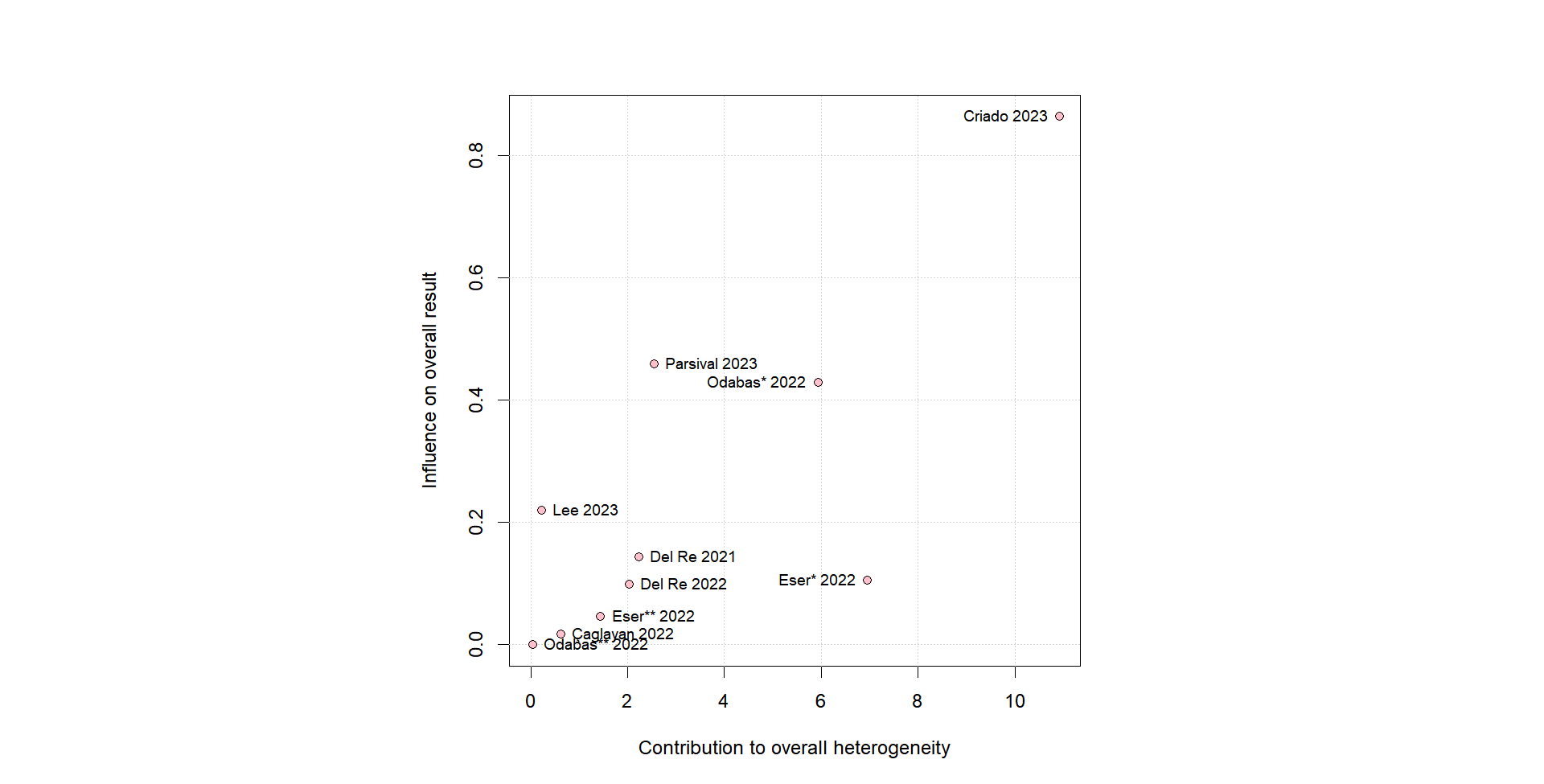


1. Funnel Plot Palbociclib


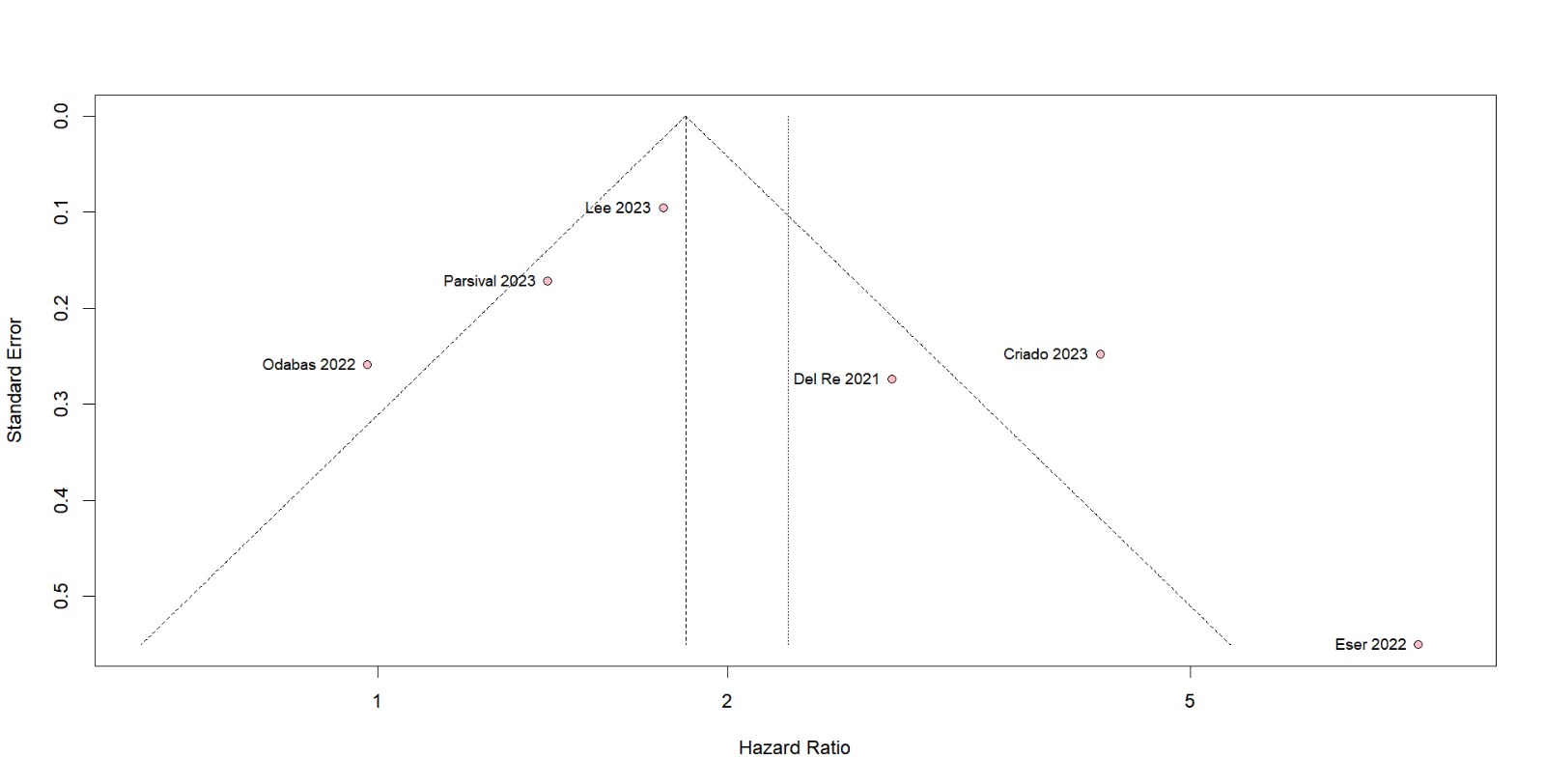


1. Funnel Plot Ribociclib


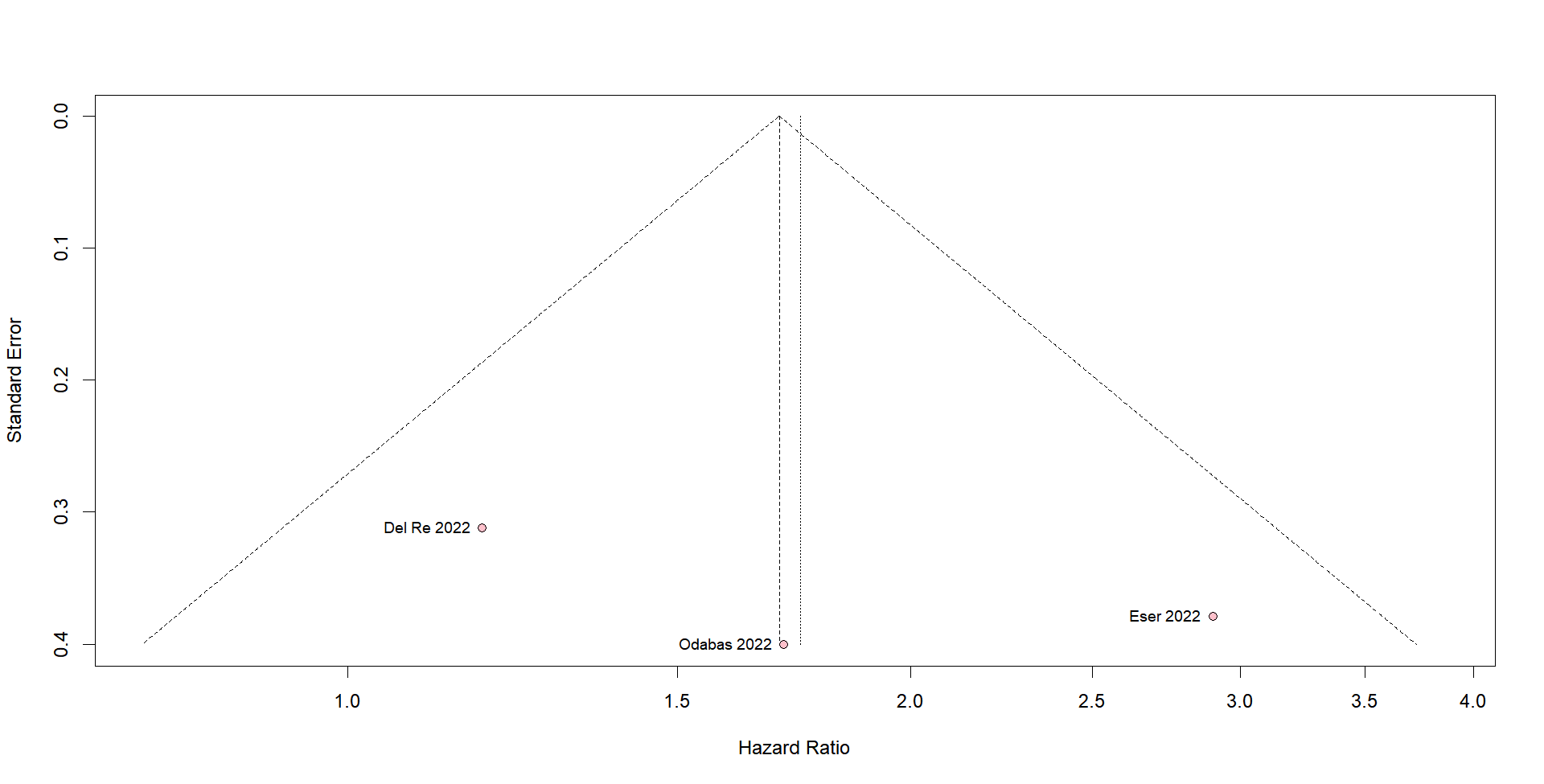


1. Baujart Ribociclib PFS

**
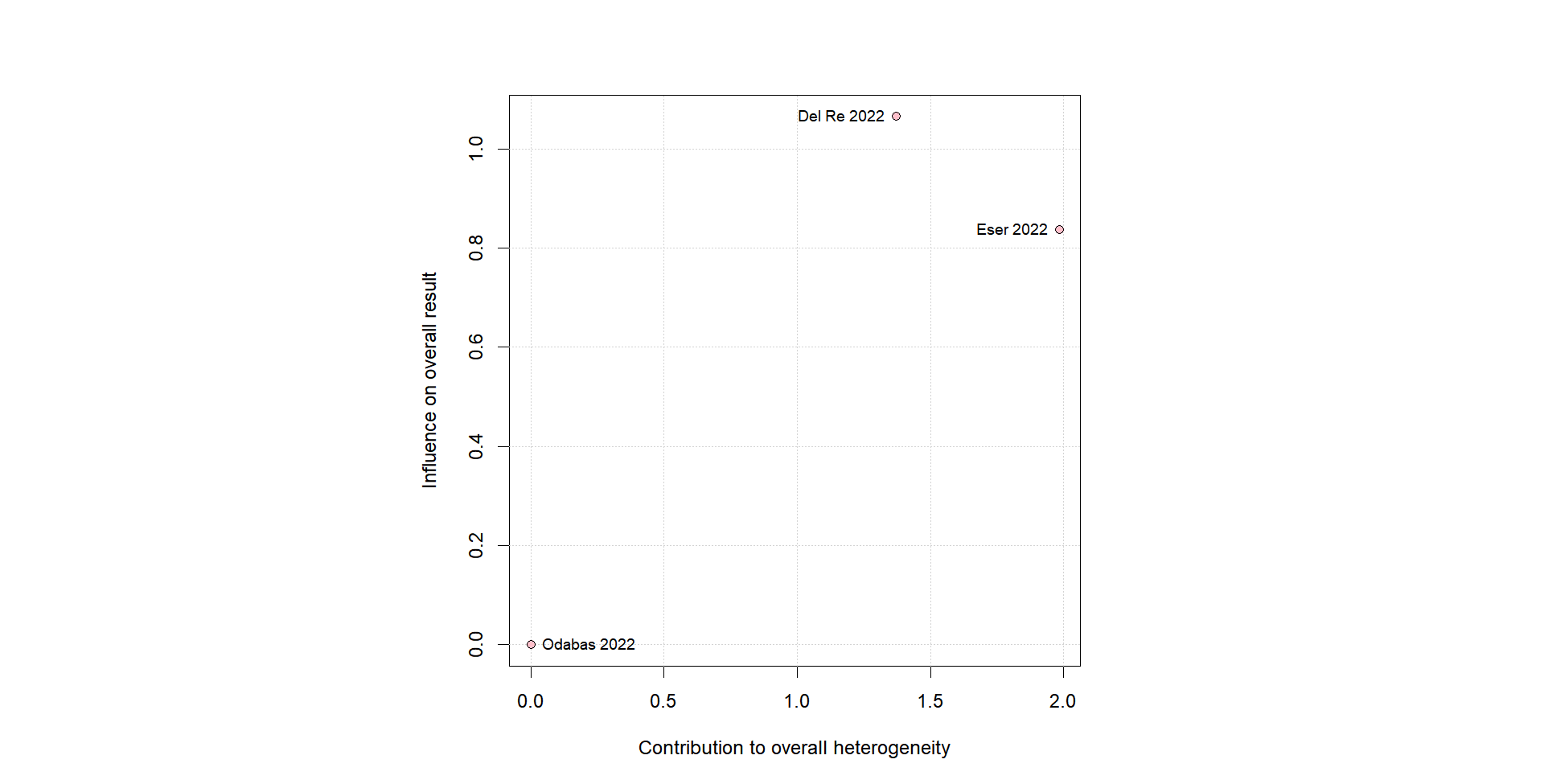
**
